# Gate-opening Induced by C8 Aromatics in a Double Diamondoid Coordination Network

**DOI:** 10.1021/acsmaterialslett.4c00511

**Published:** 2024-05-06

**Authors:** Kyriaki Koupepidou, Shi-Qiang Wang, Varvara I. Nikolayenko, Dominic C. Castell, Catiúcia R. M. O. Matos, Matthias Vandichel, Michael J. Zaworotko

**Affiliations:** † Department of Chemical Sciences, Bernal Institute, University of Limerick, Limerick V94 T9PX, Republic of Ireland; ‡ Institute of Materials Research and Engineering (IMRE), Agency for Science, Technology and Research (A*STAR), 2 Fusionopolis Way, 138634 Singapore

## Abstract

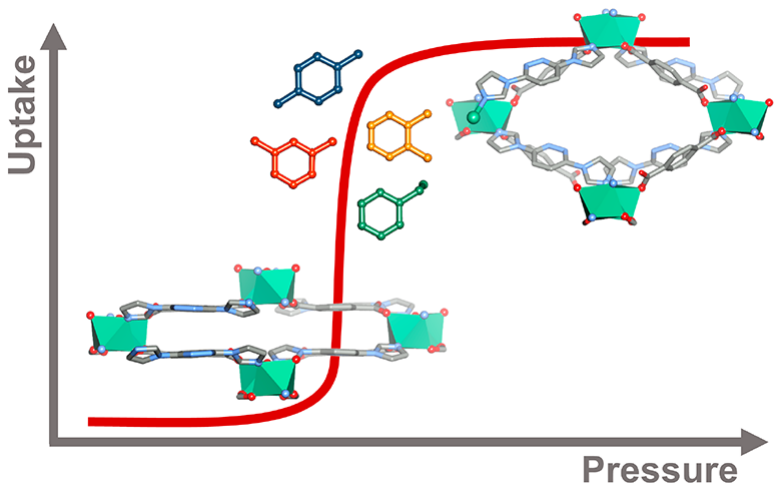

Coordination networks (CNs) that undergo guest-induced structural transformations are of topical interest thanks to their potential utility in separations and storage applications. Herein, we report a double diamondoid (**ddi**) topology CN, [Ni_2_(bimpz)_2_(bdc)_2_(H_2_O)]_*n*_ or **X-ddi-2-Ni** (H_2_bdc = 1,4-benzenedicarboxylic acid, bimpz = 3,6-bis(imidazol-1-yl)pyridazine), that undergoes structural transformations induced by C8 isomers, i.e., xylenes (*o*-xylene, OX; *m*-xylene, MX; *p*-xylene, PX) and ethylbenzene (EB). **X-ddi-2-Ni** was characterized by single-crystal to single-crystal transformations from a nonporous phase, **X-ddi-2-Ni-β**, to isostructural C8-loaded phases, namely **X-ddi-2-Ni-OX, X-ddi-2-Ni-MX, X-ddi-2-Ni-PX** and **X-ddi-2-Ni-EB**. **X-ddi-2-Ni** accommodates two C8 isomers per Ni unit, resulting in relatively high uptake (ca. 50 wt %), but with low selectivity toward C8 isomers as found using nuclear magnetic resonance (NMR) and gas chromatography (GC). In addition, a narrow range of gate-opening pressures for each isomer was determined from dynamic vapor sorption, consistent with the nonadaptable nature of the C8-loaded phase determined crystallographically, also supported by modeling.

Flexibility in coordination networks (CNs)^1−3^ is a powerful tool that can enable guest-induced tunability of pore size and/or shape through a type of induced fit binding. Flexible CNs are exemplified by those that undergo structural transformations from nonporous to porous phases through reversible “gate-opening” (or gate-closing) events. When gate-opening/closing is reversible it is of practical interest as it can mitigate some of the drawbacks of rigid CNs, e.g., by offering higher working capacity and thermal management in gas storage^4^ and separations.^5^ Such CNs tend to exhibit characteristic “S-shaped” isotherms at a threshold pressure concomitant with gate-opening.^6,7^ In addition, gas or vapor separation might be inherently more feasible in flexible CNs given that the structural transformation can depend on the guest molecule.^8,9^ In some cases, flexible CNs offer the chance for enhanced molecular recognition by adapting to a specific guest, a phenomenon described as “induced fit” that has been seen to dramatic effect with respect to the energetics of binding to acetylene.^10,11^ Further, the enthalpy values of adsorption of flexible CNs can be significantly lower than those of rigid CNs due to the phase transitions, therefore mitigating the exothermic nature of adsorption.^8^ Nevertheless, there remains a dearth of information on flexibility in the context of gas or vapor separation that is compounded because pure gas/vapor isotherms do not always translate well to multicomponent mixtures.

C8 aromatic compounds, i.e., xylenes and ethylbenzene, are of commercial value as they are involved in industrially critical synthetic processes, for which pure isomers are necessary.^12^ However, they are often byproducts in each other’s syntheses and their separation is a challenging task due to their similar physical properties and structures.^13^ The isomers are typically isolated via azeotropic distillation or fractional crystallization, which involve high cost and energy footprint. Solid–liquid or solid–vapor adsorptive separation would be expected to be advantageous in terms of energy footprint.^14,15^ Typically, in adsorptive separations there is a performance trade-off between uptake, selectivity and enthalpy.^16,17^ In C8 isomer separations, this is exemplified by materials with high selectivity but low uptake, such as Werner complexes^18,19^ or other molecular materials.^20−23^ Similarly, rigid CNs with large permanent pores such as **HKUST-1**,^24^**MOF-5**^25^ and **UiO-66**(26) achieve poor selectivities but relatively high uptake. Although this trade-off is generally observed, outliers have been reported with preferred sites for one of the isomers induced by a component of an RBB,^27^ unsaturated metal sites^28^ or through shape mismatch.^29^ The use of crystal engineering strategies involving ligand substitution^30^ or metal substitution for pore size fine-tuning^31^ can also significantly improve selectivity. Flexible CNs offer a different approach to adsorptive separations and can be advantageous compared to rigid CNs, as they can adapt to the guest through structural transformations. This can potentially enable a combination of high uptake, when the open phase is highly porous, and high selectivity, when the framework adapts to a particular guest within the mixture.^32,33^ The mechanism of flexibility toward xylenes or ethylbenzene can vary from intranetwork events (e.g., guest-responsive sites)^34^ to internetwork events (e.g., layer expansion).^35^

Based on the above ideas, in order to investigate the trade-off between uptake and selectivity, a flexible double diamondoid (**ddi**) CN, **X-ddi-2-Ni**, reported recently by our group^36^ was selected for its study toward C8 isomer sorption and separation. This was due to three primary reasons: (i) **X-ddi-2-Ni** offers a structural transformation from nonporous to porous phases, therefore promoting high working capacity; (ii) this transformation involves one of the most extreme structural changes among flexible CNs reported so far,^37−39^ resulting in a highly porous open phase and therefore promoting high uptake; (iii) the pore chemistry of the framework consists of pyridazine and benzene moieties that offer a variety of potential binding sites. The aforementioned features have potential to address uptake, selectivity and enthalpy issues that inhibit efficient C8 isomer separation. Furthermore, this represents the first study of **ddi** networks in the context of separations. Herein, we report that **X-ddi-2-Ni** undergoes single-crystal to single-crystal (SC-SC) transformations to C8-loaded phases through liquid soaking and exhibits S-shaped isotherms with respect to C8 vapors through a gate-opening mechanism. Single-crystal X-ray diffraction (SCXRD) and modelling studies were conducted to provide insight into C8 binding sites of **X-ddi-2-Ni**.

Solvothermal reaction of Ni(NO_3_)_2_, 1,4-benzendicarboxylic acid (H_2_bdc) and 3,6-bis(imidazol-1-yl)pyridazine (bimpz) yielded the as-synthesized phase, **X-ddi-2-Ni-α**.^36^ Under previously reported activation conditions,^36^ the as-synthesized phase underwent a structural transformation to a nonporous phase, **X-ddi-2-Ni-β**. Upon soaking **X-ddi-2-Ni-α** or **X-ddi-2-Ni-β** in pure *o*-xylene (OX), *m*-xylene (MX), *p*-xylene (PX) and ethylbenzene (EB), four new phases (**X-ddi-2-Ni-OX**, **X-ddi-2-Ni-MX**, **X-ddi-2-Ni-PX** and **X-ddi-2-Ni-EB**) were obtained, respectively (Figure 1). SCXRD revealed that the C8-loaded phases adopted a similar crystal packing to **X-ddi-2-Ni-α** and an equivalent normalized unit cell volume (Table S1 and Figure S1). All C8-loaded phases adopted the orthorhombic space group *Fdd*2. There were only slight changes in unit cell parameters after inclusion of the four isomers, the most pronounced differences being in the crystallographic *a* and *c* axes of the C8-loaded phases, ranging between 37.30 and 37.74 Å or 25.17 and 25.35 Å for *a* or *c* axes, respectively. This in turn resulted in different O···O distances ranging from 14.331 by 20.076 to 14.459 by 19.907 Å between the μ_2_-O atoms of different molecular building blocks (MBBs), resulting in small changes in the angles and distances that constitute the pore opening (Figure S1). The calculated and experimental powder X-ray diffraction (PXRD) patterns after soaking **X-ddi-2-Ni-α** or **X-ddi-2-Ni-β** in C8s are in good agreement, indicating bulk phase purity (Figures S2 and S3). Furthermore, soaking **X-ddi-2-Ni-β** in pure C8 isomers for 1 day resulted in shifting of PXRD peak positions to lower 2theta values (Figure 2a), indicating that a structural transformation had occurred from **X-ddi-2-Ni-β** to the OX, MX, PX and EB loaded phases. Thermogravimetric (TGA) analysis revealed that all four compounds showed a ca. 30% weight loss completed by 140 °C, corresponding to two molecules of C8 isomer per Ni unit (Figures 2b and S4–S7). This loss of C8 guest prompted us to investigate the response of **X-ddi-2-Ni-β** to C8 vapors.

**Figure 1:**
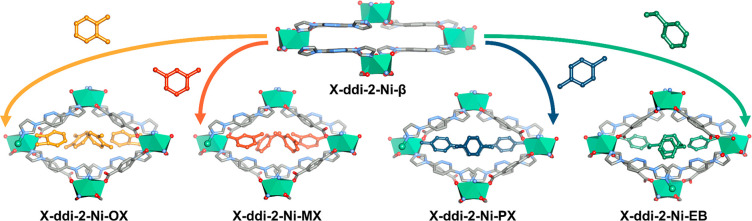
Phase transformations from **X-ddi-2-Ni-β** to **X-ddi-2-Ni-OX** (orange arrow), **X-ddi-2-Ni-MX** (red arrow), **X-ddi-2-Ni-PX** (blue arrow) and **X-ddi-2-Ni-EB** (green arrow). Hydrogen atoms are omitted for clarity. Ni_2_ units are shown in polyhedral view. Color codes: C, gray; N, blue; O, red; Ni, green. C8 guest molecules are shown in colors: OX, orange; MX, red; PX, blue; EB, green.

**Figure 2:**
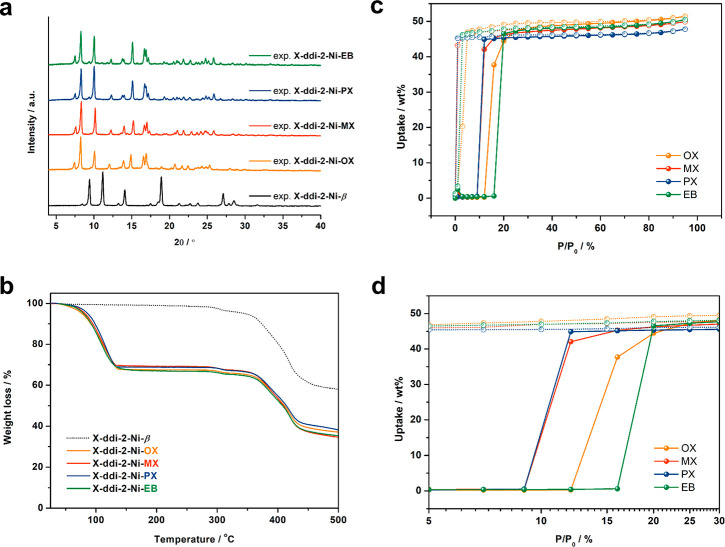
(a) Experimental (exp) PXRD patterns and (b) thermogravimetric traces of **X-ddi-2-Ni-β** (black), **X-ddi-2-Ni-OX** (orange), **X-ddi-2-Ni-MX** (red), **X-ddi-2-Ni-PX** (blue) and **X-ddi-2-Ni-EB** (green). (c) C8 aromatics vapor sorption isotherms for **X-ddi-2-Ni-β** at 298 K and (d) magnified sorption isotherms in the region of 5 to 30% P/P_0_ in logarithmic scale.

Vapor sorption isotherms were collected on **X-ddi-2-Ni-β** at 298 K (Figure 2c). No uptake was observed up to P/P_0_ ≈ 10%, after which gate-opening was observed for all four isomers. The gate-opening pressure (P_go_) values at 298 K occurred at P/P_0_ ≈ 10.4, 10.6, 14.5 and 17.9% for PX, MX, OX, and EB respectively (Figure 2d and Table S2). Uptakes of 47.8, 49.9, 51.4, and 50.5 wt % (or 4.5, 4.7, 4.8, and 4.8 mmol/g) at P/P_0_ ≈ 95% for PX, MX, OX, and EB, respectively, were observed. These uptake values are consistent with TGA results (Table S3). After desorption, the presence of **X-ddi-2-Ni-β** was confirmed by PXRD (Figure S8). Kinetic data showed that the adsorption and desorption of C8 aromatics were complete within 10 and 15 minutes, respectively (Figure S9). Pressure swing cycling experiments revealed negligible loss of performance after 10 cycles (Figure S10), while scanning electron microscopy indicated that average particle size had decreased from 84.5 × 44.5 μm^2^ to 18.4 × 10.8, 16.9 × 9.9, 18.2 × 9.8 and 17.3 × 8.9 μm^2^ after repeated cycling of OX, MX, PX and EB, respectively (10 cycles, Figure S11).

That the P_go_ values achieved through exposure to OX, MX, PX and EB vapors were distinct, although similar, prompted us to investigate and compare the respective host–guest interactions. Solvent exchange experiments were designed to obtain the crystal structures of the C8-loaded phases (see Supporting Information for experimental details). Differences in binding behavior included the number of distinct binding sites: two were located for OX and MX, but three for PX and EB (Figure 3). All four isomers exhibited one binding site in common, site I, in which the C8 isomer is “sandwiched” between two pyridazine rings of the bimpz linker. Specifically, displaced face-to-face π–π interactions of 3.53/3.82, 3.57/3.68, 3.60/3.63 and 3.70/3.77 Å were found for OX, MX, PX and EB, respectively (Table S4). Interestingly, the methyl groups formed several C–H···C van der Waals interactions with adjacent imidazole rings (Figure S12). In particular, each methyl group of OX and MX molecules interacted with imidazole rings of separate bimpz linkers, with C–H···C distances of 3.82/3.47 and 3.78/3.39 Å for OX and MX, respectively. The relatively linear shape of PX enabled each of its methyl groups to simultaneously interact with two bimpz linkers, forming a total of four C–H···C interactions (3.89/3.85 and 3.55/3.81 Å). Regarding EB, the ethyl group was disordered over two positions, leading to C–H···C interactions from both the methylene (3.68 or 3.59 Å) and methyl (3.86 or 3.49 Å) groups. Site I was found to be the main adsorptive site for all isomers, due to the strength of the face-to-face π–π interactions accommodated in this site.

**Figure 3:**
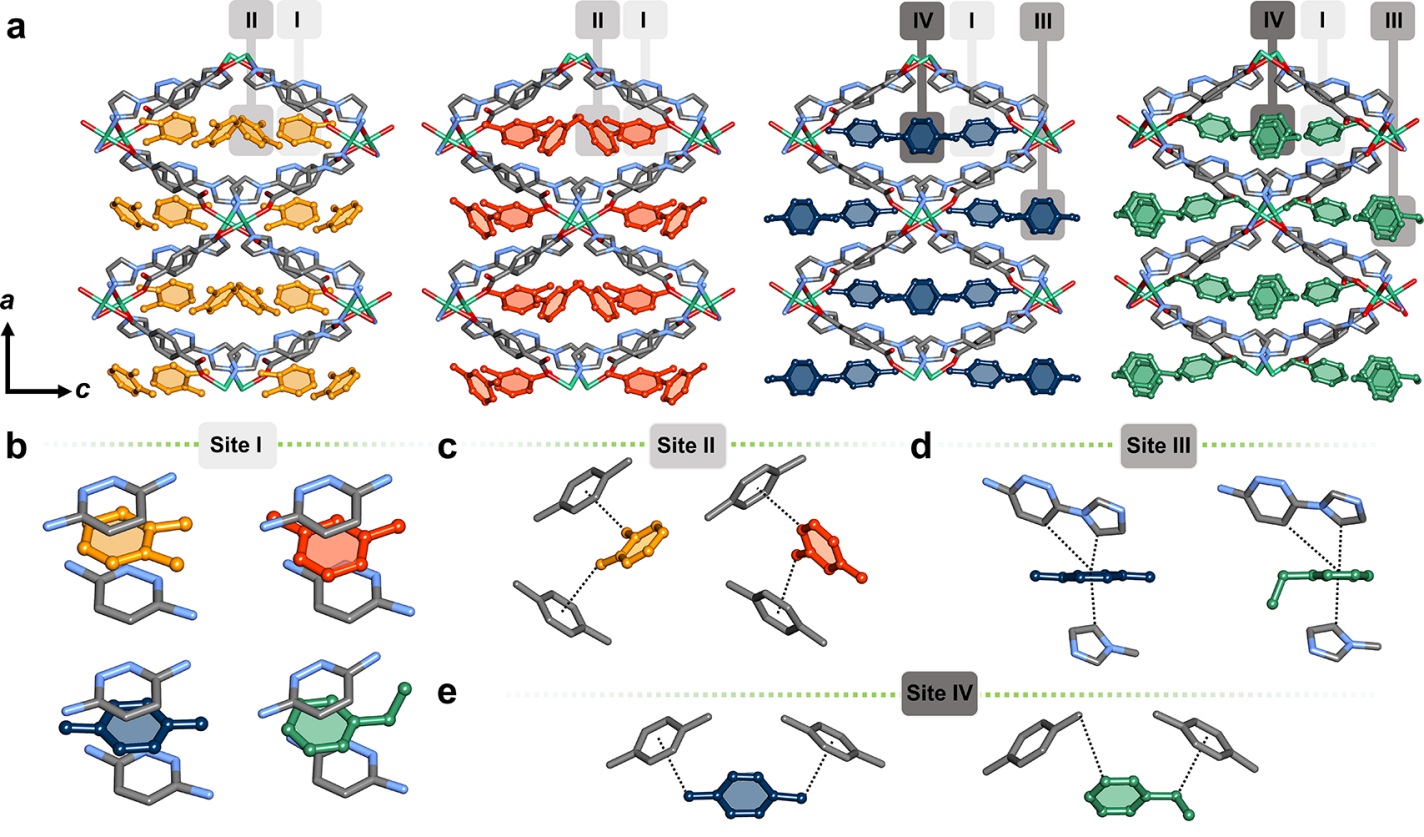
(a) Experimental* binding sites of C8 isomers in **X-ddi-2-Ni**: site I (b), site II (c), site III (d) and site IV (e). *Binding sites for EB shown at sites I, III and IV are calculated from computational studies. Hydrogen atoms are omitted for clarity. Color codes: C, gray; N, blue; O, red; Ni, green. C8 guest molecules are shown in colors: OX, orange; MX, red; PX, blue; EB, green. The ethyl group of EB at site I is disordered.

Apart from site I, differences in the remaining binding sites were evident. The second molecule of OX and MX was found at site II (Figure 3b), which is dominated by face-to-face π–π interactions (4.08 and 4.00 Å for OX and MX, respectively), alongside with C–H···π interactions from methyl groups (3.87 and 4.10 Å for OX and MX, respectively). Additionally, a neighboring imidazole ring afforded a C–H···π interaction with MX molecules, while the OX molecules were found to be positioned further away from the host in this position (Figure S13). Conversely, PX and EB were not located at site II, but occupied two additional distinct sites, III and IV (Figures 4c and 4d). The substituted methyl groups on the PX molecules were found to be engaged in host–guest interactions: C–H···C (in site III) and C–H···π (in site IV) interactions were observed (Figure S14). Due to disorder, the two remaining positions of EB could not be located crystallographically, but were modelled using computational methods (see Supporting Information for details). The positions were found to match sites III and site IV (Table S5).

**Figure 4:**
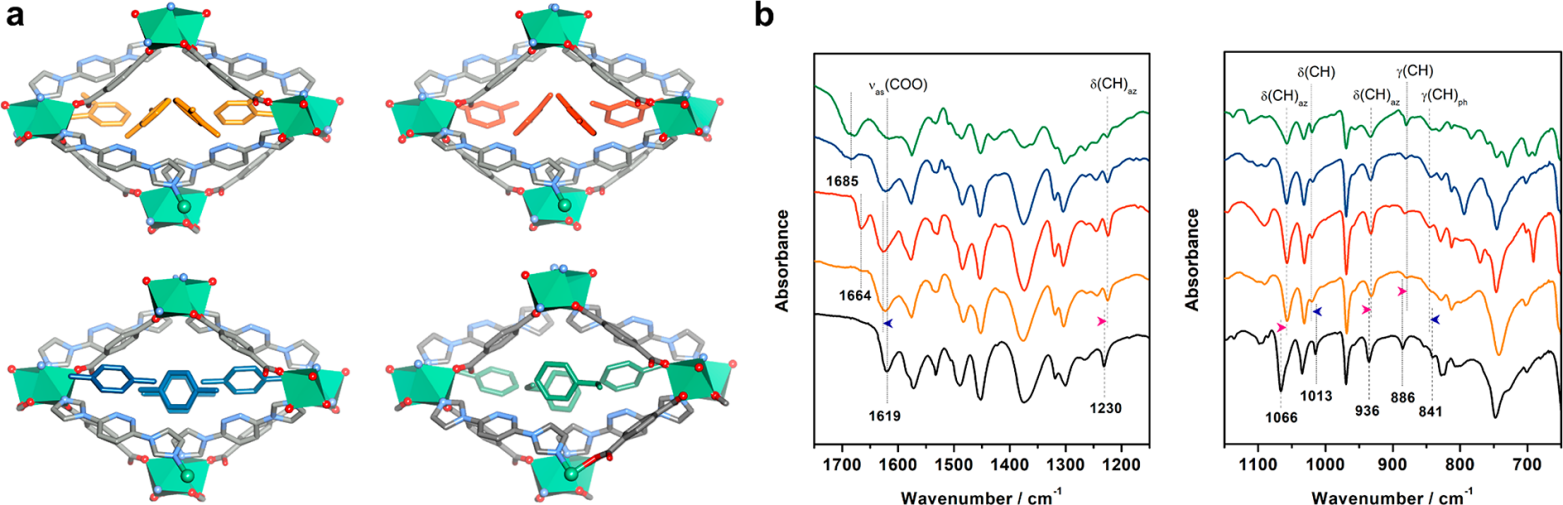
(a) Optimized binding sites of C8 isomers in **X-ddi-2-Ni**. Hydrogen atoms are omitted for clarity. Color codes: C, gray; N, blue; O, red; Ni, green. C8 guest molecules are shown in colors: OX, orange; MX, red; PX, blue; EB, green. (b) Fourier-transform infrared spectra collected for **X-ddi-2-Ni-β** (black), **X-ddi-2-Ni-2OX** (orange), **X-ddi-2-Ni-2MX** (red), **X-ddi-2-Ni-2PX** (blue) and **X-ddi-2-Ni-2EB** (green) in the region of 1750 to 1150 cm^–1^ (left) and 1150 to 650 cm^–1^ (right).

The threshold pressure or P_go_ is dependent on the energy difference between the closed and open phase framework structures.^2^ Given that the same closed phase was used to monitor the response toward all C8 guests, the underlying trend of P_go_ seen (MX ≈ PX < OX < EB) can be explained when looking at the energy of the open phase as the energetics of the loaded phases depend on supramolecular chemistry (host–guest interactions). Comparing OX and MX, MX not only exhibits stronger (shorter) interactions with the host at site I, but also forms additional C–H···π interactions at site II. Therefore, it seems intuitive that stronger and/or a larger number of interactions can stabilize the open phase by lowering its energy and the closed-to-open energy difference as reflected in a lower P_go_. Similarly, the low P_go_ for PX can be explained when examining the additional interactions C–H···C at sites I and III, as well as a C–H···N interaction at site IV, all stemming from the positioning of the methyl groups on PX. Conversely, EB achieves the highest P_go_ due to weaker C–H···C interactions at site III and the lack of appropriate shape to interact effectively at sites I and IV, as reflected in the weaker intermolecular interactions observed.

In order to gain computational insight, density functional theory (DFT) calculations were performed to optimize the positions of C8 isomers in the structures. The calculated results are consistent with the experimental data (Figures 4a and S15). The computed binding sites revealed comparable interactions with the experimentally determined sites (Tables S4 and S5, Figures S16–S19). More importantly, calculations provided information on the remaining locations of EB molecules, which overlapped with the experimental sites III and IV (Figure S15). For the average adsorption energies of the C8 isomers in their fully packed configuration (i.e., 2 C8 molecules per Ni) relative to **X-ddi-2-Ni-β**, values of −65.9, −75.8, −63.8, and −63.6 kJ/mol were determined for MX, PX, OX, and EB, respectively. These adsorption energies can be decoupled into host-framework interactions and framework-deformation energies. The framework deformation energies relative to **X-ddi-2-Ni-β** are endothermic by 46.6, 50.8, 51.7, and 57.6 kJ/mol per Ni for **X-ddi-2-Ni-MX**, **X-ddi-2-Ni-PX**, **X-ddi-2-Ni-OX**, and **X-ddi-2-Ni-EB**, respectively. This energy penalty requires exothermic host-framework interactions to open the framework in order of decreasing host-framework interaction energy (in kJ/mol), PX (−103.9) > EB (−93.4) > OX (−89.6) > MX (−89.2). While the host-framework interactions are similar for OX and MX, the framework deformation energy is notably lower for MX. PX, on the other hand, interacts stronger than MX, but needs to overcome a higher deformation barrier. The framework deformation energies are even higher in case of the OX adsorption process and the highest for EB. Summarizing, decoupling the adsorption energies in host-framework interactions and deformation barriers provided qualitative insight into understanding the previously observed sequence of P_go_.

Following experimental and computational methods to locate C8 molecules in the framework, Fourier-transform infrared spectroscopy (FTIR) was used to monitor changes upon adsorption of the isomers into **X-ddi-2-Ni-β** (Figure 4b). Analysis of the spectra obtained before and after adsorption revealed that for OX and MX a blue shift was observed in the IR band corresponding to the asymmetric stretch of the carboxylate groups (1619 cm^–1^), indicating their critical role at site II. In contrast, no such shifting was observed for PX and EB. Further, C = C aryl vibrations derived from C8 isomers can were seen around 1664 cm^–1^ for OX and MX and 1685 cm^–1^ for PX and EB. These differences match the distinct binding sites. In addition, a red shift was also observed for bands corresponding to the azole groups of the bimpz linker, namely at 1230, 1066, and 936 cm^–1^. Bands associated with the pyridazine aromatic rings (at ca. 1013, 886, and 841 cm^–1^) also shifted, indicating the involvement of these moieties in the adsorption mechanism.

The range of the host–guest interactions found in **X-ddi-2-Ni** are relatively weak compared to other reported C8 sorbents.^28^ This could indicate a potential advantage of **X-ddi-2-Ni** in terms of the energy efficiency of its regeneration. Paired with the fact that the P_go_ values differ for the isomers, we were motivated to test the separation properties of **X-ddi-2-Ni** using gas chromatography (GC) and nuclear magnetic resonance (NMR). The selectivities in binary equimolar mixtures of C8 isomers calculated from NMR were determined to be 0.97, 0.90, 1.06, 1.01, 1.06 and 1.15, for S_OX/MX_, S_OX/PX_, S_OX/EB_, S_MX/PX_, S_MX/EB_ and S_PX/EB_, respectively (Table S6 and Figures S20–S25). Similar values were obtained from GC experiments (Tables S7–S10 and Figures S26–S28). Even though subtle differences in P_go_ and binding sites indicated possible isomer recognition, the selectivity values found through binary mixtures did not suggest strong separation performance. The effect of temperature was also investigated by performing soaking experiments at 60 °C and analyzing the results through GC (Figure S29). In general, increasing the temperature had a positive effect on S_MX/EB_ and S_PX/EB_, but a negative effect on S_OX/MX_, S_OX/PX_, S_OX/EB_ and S_MX/PX_ (Table S10). Indeed, reported CNs containing large pores of similar dimensions (Table S11) also tend to suffer from weak separation performance due to insufficiently strong binding sites.^40^

When comparing CNs for adsorptive separation, two primary factors are important for overall performance: working capacity and selectivity. First, the working capacity of **X-ddi-2-Ni** is noteworthy. When compared to other flexible sorbents, **X-ddi-2-Ni** holds the second best adsorptive capacity in terms of OX, MX and PX, after **sql-1-Co-NCS** (Figures 5a and S30, Table S11).^35^ Further, **X-ddi-2-Ni** sets benchmark capacity for EB (Figure 5b). Compared to rigid sorbents reported for C8 sorption and separation (Table S12), **X-ddi-2-Ni** is second only to **MIL-101** (Figures S31–S34).^41^ While **MIL-101** is a rigid 3D compound, **sql-1-Co-NCS** is a 2D compound that undergoes layer expansion in order to accommodate a substantial amount of guest. Second, the selectivity of C8 isomers in **X-ddi-2-Ni** is poor. Various flexible sorbents that are able to adapt to one guest outperform this material in terms of selectivity.^22,35,42^ To rationalize this, we refer to the open phase of **X-ddi-2-Ni** which is isostructural for all isomers (Table S1). The above observations underscore that separations in flexible CNs generally occur in sorbents that can adapt their structure to different guests, which represents an important design principle in terms of designing flexible sorbents for separations.

**Figure 5:**
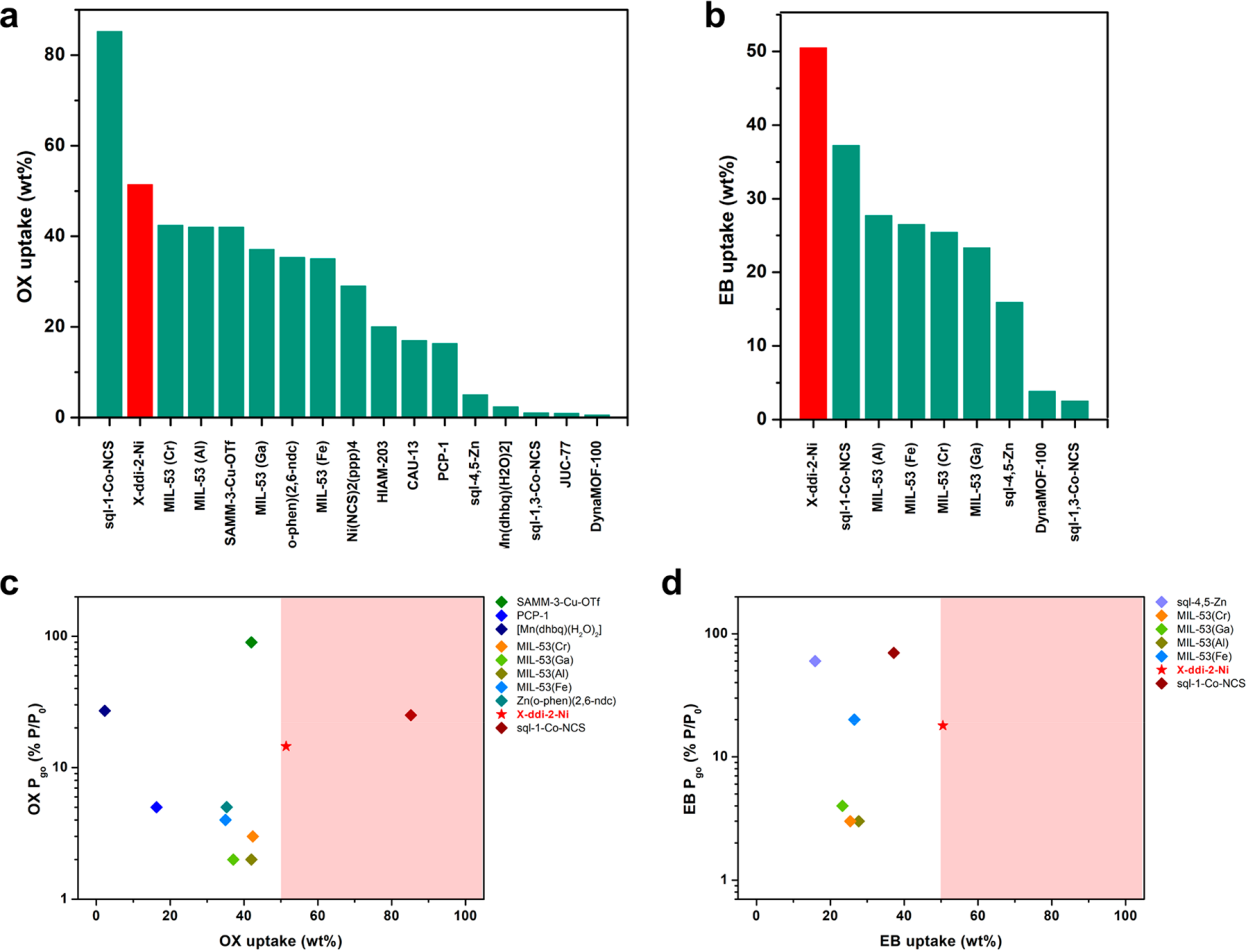
Comparison of uptake of (a) OX and (b) EB for **X-ddi-2-Ni** (red) and other flexible sorbents (green). P_go_ values plotted against uptake of (c) OX and (d) EB for **X-ddi-2-Ni** and other flexible sorbents. Red shaded area represents the area of high adsorptive capacity (≥50 wt %).

Further, in terms of flexible sorbents, the dearth of information on their ability to sorb and/or separate C8 aromatics is apparent (Table S11). Even when relevant separation studies are reported, the isotherms are often omitted,^18,29,43,44^ making it difficult to extrapolate structure–property relationships relevant to P_go_ values. Where P_go_ is available, comparison with other sorbents suggests that **X-ddi-2-Ni** lies in the middle of the performance spectrum, with an intermediate P_go_ compared to other sorbents, but one of the highest (in the case of EB the highest) loading capacities (Figures 5c–d). In some cases, even small differences in P_go_ (within the range of 2% P/P_0_)^32^ can result in separation performance, however, different open phases were observed, further supporting the conclusions derived herein.

In conclusion, this work offers insights into structure–function relationships in flexible sorbents, both generally and in the context of C8 aromatics sorption and separation. In particular, that the C8-loaded phases of **X-ddi-2-Ni** are structurally similar to each other is reflected in the narrow range of P_go_ values and low C8 selectivity. Overall, whereas **X-ddi-2-Ni** is flexible, it is not adaptable to C8 isomers in a manner similar to that seen for leading C8 sorbents, some of which, as detailed in the introduction, can be described as examples of induced fit binding. This work therefore highlights that the trade-off between uptake and selectivity typical of rigid sorbents can also occur in flexible sorbents with large pore phases that are not adaptable.

## Supporting Information Available

Materials and methods, supporting figures and supporting tables (PDF)
**X-ddi-2-Ni-OX_optimized** (CIF)
**X-ddi-2-Ni-MX_optimized** (CIF)
**X-ddi-2-Ni-PX_optimized** (CIF)
**X-ddi-2-Ni-EB_optimized** (CIF)

## Supplementary Material

tz4c00511_si_001.pdf

tz4c00511_si_002.cif

tz4c00511_si_003.cif

tz4c00511_si_004.cif

tz4c00511_si_005.cif
