# Supplementary material for: Gate-opening Induced by C8 Aromatics in a Double Diamondoid Coordination Network
Source: ACS Mater Lett. 2024 May 6;6(6):2197–204. doi: 10.1021/acsmaterialslett.4c00511 (PMC11151277; doi:10.1021/acsmaterialslett.4c00511)
Supplement: Supplementary file 1 — tz4c00511_si_001.pdf [file tz4c00511_si_001.pdf]

Gate-opening Induced by C8 Aromatics in a Double Diamondoid Coordination Network

Kyriaki Koupepidou,[a] Shi-Qiang Wang,[a,b] Varvara I. Nikolayenko,[a] Dominic C. Castell,[a] Catiúcia R. M. O. Matos,[a] Matthias Vandichel[a] and Michael J. Zaworotko[a,*]

[a]Department of Chemical Sciences, Bernal Institute, University of Limerick, Limerick V94 T9PX, Republic of Ireland.

[b]Institute of Materials Research and Engineering (IMRE), Agency for Science, Technology and Research (A*STAR), 2 Fusionopolis Way, 138634 Singapore.

S1. Methods

S1.1. Materials and Synthesis

The linker 3,6-bis(imidazol-1-yl)pyridazine (bimpz) were synthesized according to previously reported procedures.[1] Other reagents and solvents were commercially available and were used without further purification.

Synthesis of X-ddi-1-Ni-α ([Ni_2(bimpz)$_2$(bdc)$_2$(H_2O)]·6DMF). A mixture of $Ni(NO_3)_2·6H_2O$ (29 mg, 0.1 mmol), H_2bdc (17 mg, 0.1 mmol), bimpz (11 mg, 0.05 mmol) and DMF (10 mL) was added to a 28-mL glass vial. The vial was capped tightly, ultrasonicated for 5 minutes and then placed in an oven at 105 °C. After 24 hours, the vial was removed from the oven and allowed to cool to room temperature. Green block shaped crystals were harvested by filtration and washed with DMF. Yield: 65%.

Synthesis of X-ddi-2-Ni-β ([Ni_2(bimpz)$_2$(bdc)$_2$(H_2O)]). The closed phase **X-ddi-2-Ni-β** was obtained by heating the α phase at 105 °C under vacuum for 12 h.

Synthesis of X-ddi-2-Ni-OX ([Ni_2(bimpz)$_2$(bdc)$_2$(H_2O)]·4OX). The OX-loaded phase **X-ddi-2-Ni-OX** was obtained by soaking the **β** phase in OX for 1 day, or performing solvent exchange of α phase at 60 °C throughout 3 days. In the case of obtaining from **X-ddi-2-Ni-OX** from **α**, the solvent was replaced with fresh OX once per day and the mixture was heated at 60 °C to assist the solvent exchange process.

Synthesis of X-ddi-2-Ni-MX ([Ni_2(bimpz)$_2$(bdc)$_2$(H_2O)]·4MX). The MX-loaded phase **X-ddi-2-Ni-MX** was obtained by similar methods as described for **X-ddi-2-Ni-OX**, by replacing OX with MX.

Synthesis of X-ddi-2-Ni-PX ([Ni_2(bimpz)$_2$(bdc)$_2$(H_2O)]·4PX). The PX-loaded phase **X-ddi-2-Ni-PX** was obtained by similar methods as described for **X-ddi-2-Ni-OX**, by replacing OX with PX.

Synthesis of X-ddi-2-Ni-EB ([Ni_2(bimpz)$_2$(bdc)$_2$(H_2O)]·4EB). The PX-loaded phase **X-ddi-2-Ni-EB** was obtained by similar methods as described for **X-ddi-2-Ni-OX**, by replacing OX with EB.

Bulk synthesis. Bulk synthesis of **X-ddi-2-Ni-α** was performed by scaling up the crystallization reagents and solvent 10 times. The large batches were used for activation, SEM analysis, sorption measurements, and miscellaneous characterization.

S1.2. Single-crystal X-ray Diffraction Measurements

S1.2.1. General Procedure

Suitable single crystals of **X-ddi-2-Ni-OX**, **X-ddi-2-Ni-MX**, **X-ddi-2-Ni-PX** and **X-ddi-2-Ni-EB** were chosen for single-crystal X-ray diffraction measurements. Diffraction data for were collected at 100 K on a Bruker D8 Quest diffractometer equipped with a CuKα microfocus source (λ = 1.5406 Å) and a Photon

100 detector. In all cases, data was indexed, integrated and scaled in APEX4.[2] Absorption correction was performed by multi-scan method using in SADABS.[3] Space group determination was performed simultaneously with structure solution using SHELXT intrinsic phasing methods and the solution was refined on F2 using SHELXL non-linear least squares implemented in Olex2 v1.2.10.[4] Anisotropic thermal parameters were applied to all framework non-hydrogen atoms. All the hydrogen atoms were generated geometrically. Where noted, the data was treated with the SQUEEZE routine[5] using the solvent masking feature of Olex2. X-ray experimental data and refinement parameters are given in Table S1. The crystal structures have been deposited in the Cambridge Crystallographic Data Centre (CCDC 2339071-2339074).

S1.2.2. Specific Refinement Details

The structures reported in this manuscript are all metal-organic framework structures with bridging water molecules between two heavy atoms, i.e. Ni^{2+} centers. As a result, the hydrogen atoms on the bridging oxygen are not detected. Additionally, the crystals of the **X-ddi** materials are merohedral twins, so the appropriate twin law refinement was performed on the raw data.

Specific Details and Alerts:

X-ddi-2-Ni-OX. Two positions of OX molecules were modelled per Ni unit, with occupancies of 0.748 and 0.862. Further, the aromatic ring of the bdc^{2-} linker was found to be disordered over two positions with partial occupancies of 0.512 and 0.488. The carboxylic group of the same linker was found to be disordered over two positions with partial occupancies of 0.598 and 0.402. The OX molecules were refined isotropically due to high degree of disorder, since the guest molecules are not strongly bound as suggested by experimental data. The rest of the atoms were refined anisotropically.

PLAT341_ALERT_3_B Low Bond Precision on C-C Bonds .... 0.02429 Ang.

Response: X-ddi-2-Ni consists of large pores in which C8 aromatic molecules can be found. Even though their locations can be modelled, they exhibit high degree of motion due to the fact that they are not strongly bound to the pore walls through supramolecular interactions. This is reflected in a B level alert regarding low bond precision of the C-C bonds in the disordered guest molecules.

PLAT601_ALERT_2_B Unit Cell Contains Solvent Accessible VOIDS of . 115 Ang**3

Response: X-ddi-2-Ni consists of large pores in which C8 aromatic molecules can be found. Two molecules of o-xylene were modelled in the pores, resulting in efficient packing. The remaining electron density amounting to less than 0.2 of a C8 molecule could be attributed to the partial occupancies in the measured crystal.

X-ddi-2-Ni-MX. Two positions of MX molecules were modelled per Ni unit, out of which one had full occupancy and the other had an occupancy of 0.580. Further, the aromatic ring of the bdc^{2-} linker was found to be disordered over two positions with partial occupancies of 0.580 and 0.420. The MX molecules were refined isotropically due to high degree of disorder, since the guest molecules are not strongly bound as suggested by experimental data. The rest of the atoms were refined anisotropically.

<u>PLAT341_ALERT_3_B</u> Low Bond Precision on C-C Bonds .... 0.01967 Ang.

Response: X-ddi-2-Ni consists of large pores in which C8 aromatic molecules can be found. Even though their locations can be modelled, they exhibit high degree of motion due to the fact that they are not strongly bound to the pore walls through supramolecular interactions. This is reflected in a B level alert regarding low bond precision of the C-C bonds in the disordered guest molecules.

<u>PLAT601_ALERT_2_B</u> Unit Cell Contains Solvent Accessible VOIDS of . 123 Ang**3

Response: X-ddi-2-Ni consists of large pores in which C8 aromatic molecules can be found. Two molecules of m-xylene were modelled in the pores, resulting in efficient packing. The remaining electron density amounting to less than 0.2 of a C8 molecule could be attributed to the partial occupancies in the measured crystal.

X-ddi-2-Ni-PX. Three positions of PX molecules were modelled per Ni unit, out of which two were found in special positions therefore only containing half the PX molecule. Full occupancy for the full PX site was found, while one of the half PX sites was found in full occupancy and the other at 0.720, amounting to a total of 1.860 PX per Ni unit. Further, the aromatic ring of the bdc^{2-} linker was found to be disordered over two positions with partial occupancies of 0.575 and 0.425. The PX molecules were refined isotropically due to high degree of disorder, since the guest molecules are not strongly bound as suggested by experimental data. The rest of the atoms were refined anisotropically.

<u>PLAT341_ALERT_3_B</u> Low Bond Precision on C-C Bonds .... 0.0229 Ang.

Response: X-ddi-2-Ni consists of large pores in which C8 aromatic molecules can be found. Even though their locations can be modelled, they exhibit high degree of motion due to the fact that they are not strongly bound to the pore walls through supramolecular interactions. This is reflected in a B level alert regarding low bond precision of the C-C bonds in the disordered guest molecules.

X-ddi-2-Ni-EB. One position of EB molecules was modelled per Ni unit with occupancy of 0.956. The ethyl group of the EB molecule could not be refined with the correct geometry due to disorder. The position of the ethyl group is better reflected in the computational segment of the manuscript. The rest of the EB molecules were accounted for using the SQUEEZE routine as implemented in Olex2. The solvent mask calculation amounted to 696 electrons in per unit cell, which is consistent with the presence of

0.75[C8H10] per formula unit. Further, the aromatic ring of the bdc^{2-} linker was found to be disordered over two positions with partial occupancies of 0.560 and 0.440. The EB molecules were refined isotropically due to high degree of disorder, since the guest molecules are not strongly bound as suggested by experimental data. The rest of the atoms were refined anisotropically.

S1.3. Powder X-ray Diffraction Measurements

Powder X-ray diffraction data was collected on crushed microcrystalline samples. Diffractograms were recorded using a PANalytical Empyrean™ diffractometer equipped with a PIXcel3D detector, operating in scanning line detector mode with an active length of 4 utilizing 255 channels, in the Continuous Scanning mode with the goniometer in the theta-theta orientation. The diffractometer is fitted with an Empyrean Cu LFF (long fine-focus) HR (9430 033 7310x) tube operated at 40 kV and 40 mA, and CuKα radiation (λα = 1.540598 Å) was used for diffraction experiments. Incident beam optics included the Fixed Divergences slit with anti-scatter slit PreFIX module, with a 1/8° divergence slit and a 1/4° anti-scatter slit, as well as a 10 mm fixed incident beam mask and a Soller slit (0.04 rad). Divergent beam optics included a P7.5 anti-scatter slit, a Soller slit (0.04 rad), and a Ni-β filter. The data was collected from 5°-40° (2θ) with a step-size of 0.016413° and a varied scan time of 30-200 seconds per step.

S1.4. Thermogravimetric Analysis (TGA)

Thermogravimetric analysis (TGA) was performed using a TA Instruments Q50 system. Samples were loaded into aluminium sample pans and heated at 10 °C/min from room temperature to 550 °C under N_2 flow.

S1.5. Fourier-Transform Infrared (FTIR) Spectroscopy

Spectra were obtained by using a Perkin Elmer Spectrum 100 FTIR Spectrometer with ATR and Spotlight 200 FTIR microscope attachment. A small amount of powder microcrystalline sample was placed onto the ATR plate and the spectra were collected in the range of 4000-650 cm^{-1} with resolution of 0.5 cm^{-1}.

S1.6. Vacuum Dynamic Vapor Sorption

Dynamic vapor sorption measurements were conducted using a Surface Measurement Systems DVS Vacuum at 298 K. Samples of **X-ddi-2-Ni** were further degassed under high vacuum ($1x10^{-4}$ Torr) *in-situ* and stepwise increase in relative pressure were controlled by equilibrated weight changes of the sample (dM/dT = 0.01%/min) from 0 to 95%. Vacuum pressure transducers were used with ability to measure from $1x10^{-6}$ to 760 Torr with a resolution of 0.01%. Approximately 10 mg of sample was used for each experiment (9.8007 mg for OX, 7.7403 mg for MX, 15.1352 mg for PX and 5.9578 mg for EB). The mass of the sample was determined by comparison to an empty reference pan and recorded by a high resolution microbalance with a precision of 0.1 μg. For adsorption-desorption recyclability tests (Figure S14),

sample of **X-ddi-2-Ni** was first degassed and then the sample was exposed to 90% relative pressure of C8 isomer for 1 hour as the adsorption step; after that the sample was desorbed at 40°C under vacuum for 1 hour and just under vacuum for half hour as the desorption step. The adsorption and desorption steps were repeated up to ten times.

S1.7. Nuclear Magnetic Resonance (NMR) Spectroscopy

In each experiment, about 20 mg samples of **X-ddi-2-Ni-β** were immersed in 3 mL of equimolar binary mixture of C8 aromatics for two days. PXRD patterns confirmed that **X-ddi-2-Ni-β** can transform to the C8-loaded phases within one day (Figure S3). The saturated samples were filtered and air-dried (for *ca.* 5 minutes) under ambient conditions (*ca.* 20 °C) to remove xylenes adhering to the surface. The samples were then soaked in 2 mL $CDCl_3$ for three days. The supernatant of each samples were collected to measure 1H NMR spectra using a JEOL ECX400 NMR spectrometer.

The NMR spectra were analysed to calculate the selectivity as follows:

$$S_{ij} = \frac{x_i}{x_j}\frac{y_j}{y_i}$$

Where S is the selectivity of component i relative to component j, x_i and x_j are the mole fractions of components i and j in the adsorbed phase, and y_i and y_j are the mole fractions of components i and j in the liquid phase.

The ratio of x_i / x_j can be derived from the integrated area ratio of corresponding methyl groups or methylene group of C8 aromatics in NMR spectra. When component i and j are both xylene isomers, the selectivity is defined as:

$$S_{ij} = \frac{x_i}{x_j}\frac{y_j}{y_i} = \frac{q_i}{q_j}\frac{y_j}{y_i}$$

Where q_i and q_j are the relatively integrated area of corresponding methyl groups of xylene isomers. When component i is one of xylene isomers while j is ethylbenzene, the selectivity is defined as:

$$S_{ij} = \frac{x_i}{x_j}\frac{y_j}{y_i} = \frac{q_i}{3 * q_j}\frac{y_j}{y_i}$$

Where q_i is the relatively integrated area of corresponding methyl groups (including 6 H) of xylene isomers, while q_j is the relatively integrated area of corresponding methylene group (including 2 H) of ethylbenzene.

S1.8. Gas Chromatography (GC) Analysis

The GC analyses were carried out on an Agilent 6890A gas chromatograph fitted with a 7683B ALS (automated liquid sampler) equipped with a flame ionization detector (FID). The column used was an Agilent DB-Wax (Length: 30 m, Inner diameter: 320 µm, Film thickness: 0.25 µm). An initial temperature of 40 °C and initial hold time of two minutes were used with a ramp rate of 10 °C · min^{-1} to a maximum temperature of 180 °C. The injector and detector were kept at 220 °C and nitrogen was used as carrier gas with a flow rate of 1 ml · min^{-1}. 1 µL of each liquid sample was injected through the GC inlet with a split ratio of 100:1 and a split flow rate of 142.45 ml · min^{-1}. Dichloromethane HPLC/GC grade 99.9% (Sigma-Aldrich) was used as eluent and solvent for the standard solutions of 500 ppm of all the C8 aromatic isomers and the samples of **X-ddi-2-Ni**. With the method described above, we could obtain separate retention times for PX, MX, OX and EB.

In a typical experiment, 20 mg samples of **X-ddi-2-Ni** were immersed in equimolar (2 g each) binary liquid of C8 aromatics at room temperature and at 60 °C for three days. After complete uptake, samples were filtered and air-dried (*ca.* 30 minutes) under ambient conditions (*ca.* 25 °C) to remove xylenes adhering to the surface of samples. After that, the samples were soaked in 2 mL CH_2Cl_2 (HPLC/GC grade 99.9%, Sigma-Aldrich) for three days, to allow complete extraction of C8 aromatics from the solid phase to the solution. Aliquots of 1 mL of the extracted C8s in CH_2Cl_2 were collected for GC measurements without dilution to obtain the selectivity coefficients. The peak areas of individual C8 aromatics shown in chromatograms were used to calculate the selectivity coefficients. The binary solutions of the xylenes were also analysed to obtain the molar ratio of each xylene in the mixture. The selectivity coefficient of component i relative to component j is expressed as:

$$S_{ij} = \frac{x_i}{x_j}\frac{y_j}{y_i}$$

Where S is the selectivity of component i relative to component j, x_i and x_j are the mole fractions of components i and j in the adsorbed phase, and y_i and y_j are the mole fractions of components i and j in the liquid phase.

S1.9. Scanning Electron Microscopy (SEM)

Scanning electron microscopy measurements were carried out for the activated samples to investigate particle size. The images were collected on a Hitachi SU-70 instrument, using 3 kV acceleration voltage and a working distance of 15 mm. Before the measurement, the samples were dispersed on carbon tape attached to SEM stubs, and were gold-coated for 50 seconds to enhance surface conductivity.

S1.10. Computational Studies

Periodic Density Functional Theory (DFT) calculations were performed using the projected augmented wave (PAW) formalism[6] as implemented in the Vienna Ab Initio Simulation Package (VASP 5.4.4),[7, 8] employing the BEEF-vdW exchange-correlation functional.[9] The atomic positions of primitive unit cells obtained from experimentally refined structures (see Table S1) were optimized, using the conjugate gradient algorithm with force and electronic convergence criteria of 0.02 eV/Å and 10^{-6} eV, a Gaussian smearing of 0.02 eV, an energy cutoff of 550 eV, and Monkhorst-Pack[10] k-point meshes of 2x2x2 for all four **X-ddi-2-Ni-C8**, and 2x3x2 for **X-ddi-2-Ni-β**. Spin-polarized calculations were performed with two unpaired electrons per Ni. After optimization, the average adsorption energy was calculated for each C8 isomer with respect to the closed form (**X-ddi-2-Ni-β**) and the C8 isomer optimized in a 20x20x20 $Å^3$ periodic box. To provide insight into the framework deformation energy for each of the four optimized **X-ddi-2-Ni-C8** structures, the C8 isomers were cut, and a single point energy calculation was performed on the remaining open empty host structure (**X-ddi-2-Ni-C8-empty**). This resulted in deformation energies of 46.6, 50.8, 51.7, and 57.6 kJ/mol per Ni for **X-ddi-2-Ni-MX**, **X-ddi-2-Ni-PX**, **X-ddi-2-Ni-OX**, and **X-ddi-2-Ni-EB**, respectively, and exotherm host-framework interaction energies (in kJ/mol per C8), PX (-103.9) > EB (-93.4) > OX (-89.6) > MX (-89.2). Both are contributing to the net adsorption energies (in kJ/mol per C8) calculated relative to **X-ddi-2-Ni-β,** MX (-65.9), PX (-75.8), OX (-63.8), and EB (-63.6).

S2. Figures and Tables

Table S1. Crystallographic data and refinement parameters for **X-ddi-2-Ni** (part 1).

Compound	X-ddi-2-Ni-α	X-ddi-2-Ni-β
Formula	$[C_{36}H_{24}N_{12}Ni_2O_9][5.53(C_3H_7NO)]$	$C_{36}H_{24}N_{12}Ni_2O_9$
Formula	1290.31	886.09
Temperature	100(2)	100(2)
Crystal system	Monoclinic	Orthorhombic
Space group	*Cc*	*Fdd*2
a (Å)	13.574(3)	14.186(4)
b (Å)	37.658(5)	41.627(3)
c (Å)	14.199(3)	12.854(4)
α (°)	90	90
β (°)	117.95(4)	90
γ (°)	90	90
V (Å^3)	6411.6(2)	7591.1(10)
Z	4	8
D_c (g·cm^{-3})	1.337	1.551
μ (mm^{-1})	1.356	1.853
R_{int}	0.0417	0.0573
GOF	1.043	1.065
R_1 [I > 2σ (I)]	0.0416	0.0812
WR_2 [all data]	0.1144	0.2576
Diff peak /	1.151 / -0.585	1.403 / -0.804
Flack	0.03(2)	0.06(2)
No. CCDC	2234140	2234141

$R_1 = \Sigma||F_o| - |F_c||/\Sigma|F_o|$. $wR_2 = [\Sigma w(F_o^2 - F_c^2)^2 / \Sigma w(F_o^2)^2]^{½}$

Table S1. Crystallographic data and refinement parameters for **X-ddi-2-Ni** (part 2).

Compound	X-ddi-2-Ni-OX	X-ddi-2-Ni-MX
Formula	$[C_{18}H_{12}N_6NiO_{4.5}][1.61(C_8H_{10})]$	$[C_{18}H_{12}N_6NiO_{4.5}][1.577(C_8H_{10})]$
Formula weight	613.62	610.47
Temperature (K)	100.00	100.00
Crystal system	Orthorhombic	Orthorhombic
Space group	*Fdd2*	*Fdd2*
a (Å)	37.2998(6)	37.4785(6)
b (Å)	13.9222(2)	13.7359(2)
c (Å)	25.3460(4)	25.3328(4)
α (°)	90	90
β (°)	90	90
γ (°)	90	90
V ($Å^3$)	13162.1(4)	13041.3(3)
Z	16	16
D_c ($g·cm^{-3}$)	1.239	1.244
μ (mm^{-1})	1.211	1.219
R_{int}	0.0390	0.0443
GOF	1.077	1.043
R_1 [$I > 2σ(I)$]	0.0898	0.0903
WR_2 [all data]	0.2539	0.2583
Diff peak / hole (e $Å^{-3}$)	1.391 / -0.915	1.069 / -1.138
Flack	0.07(8)	0.07(7)
No. CCDC	2339071	2339072

$R_1 = \Sigma||F_o| - |F_c||/\Sigma|F_o|$. $wR_2 = [\Sigma w(F_o^2 - F_c^2)^2 / \Sigma w(F_o^2)^2]^{1/2}$

Table S1. Crystallographic data and refinement parameters for **X-ddi-2-Ni** (part 3).

Compound	X-ddi-2-Ni-PX	X-ddi-2-Ni-EB
Formula	$[C_{18}H_{12}N_6NiO_{4.5}][3.718(C_4H_5)]$	$[C_{18}H_{12}N_6NiO_{4.5}][0.956(C_8H_{10})][+solvent]$
Formula weight	640.39	541.98
Temperature (K)	100.00	150.00
Crystal system	Orthorhombic	Orthorhombic
Space group	*Fdd*2	*Fdd*2
a (Å)	37.7411(5)	37.627(4)
b (Å)	13.7030(2)	13.7893(13)
c (Å)	25.1739(4)	25.214(3)
α (°)	90	90
β (°)	90	90
γ (°)	90	90
V ($Å^3$)	13019.1(3)	13082(2)
Z	16	16
D_c ($g{\cdot}cm^{-3}$)	1.307	1.106
μ (mm^{-1})	1.246	1.160
R_{int}	0.0514	0.0285
GOF	1.061	1.056
R_1 [$I > 2\sigma(I)$]	0.0812	0.0513
WR_2 [all data]	0.2171	0.1429
Diff peak / hole (e $Å^{-3}$)	1.279 / -1.195	0.548 / -0.523
Flack	0.08(7)	0.15(5)
No. CCDC	2339073	2339074

$R_1 = \Sigma||F_o| - |F_c||/\Sigma|F_o|$. $wR_2 = [\Sigma w(F_o^2 - F_c^2)^2 / \Sigma w(F_o^2)^2]^{½}$

Table S1. Crystallographic data and refinement parameters for **X-ddi-2-Ni** (part 4).

**Compound**	**X-ddi-2-Ni-OX-optimized (computational)**	**X-ddi-2-Ni-MX-optimized (computational)**	**X-ddi-2-Ni-PX-optimized (computational)**	**X-ddi-2-Ni-EB-optimized (computational)**
Space group	P1	P1	P1	P1
a (Å)	14.459	14.4086	14.3309	14.3692
b (Å)	22.5483	22.6185	22.6832	22.6469
c (Å)	19.9067	19.9582	20.0759	20.0371
α (°)	39.2047	38.9319	38.5588	38.7393
β (°)	80.308	80.5593	80.6094	80.4967
γ (°)	60.4873	60.5087	60.8318	60.764
V ($Å^3$)	3290.53	3260.35	3254.78	3270.58

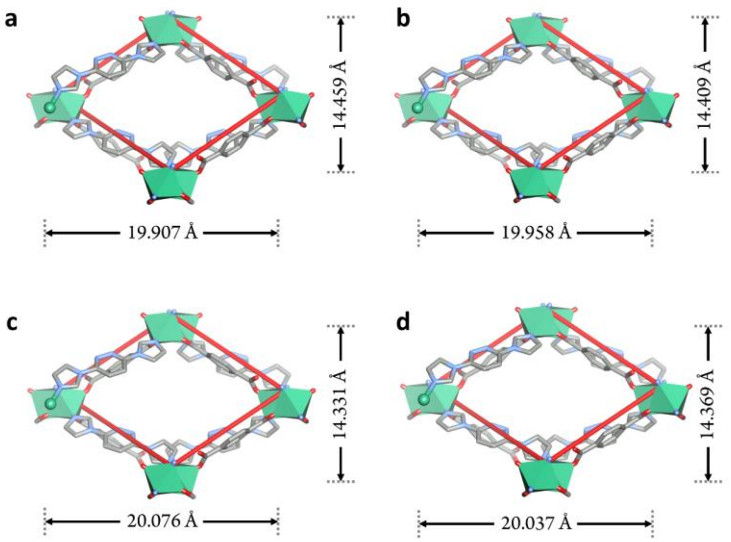

Figure S1. Crystal packing of (a) **X-ddi-2-Ni-OX**, (b) **X-ddi-2-Ni-MX**, (c) **X-ddi-2-Ni-PX** and (d) **X-ddi-2-Ni-EB** and distances that constitute the pore opening. Red lines connect the μ_2-O atoms of each MBB. Hydrogen atoms are omitted for clarity.

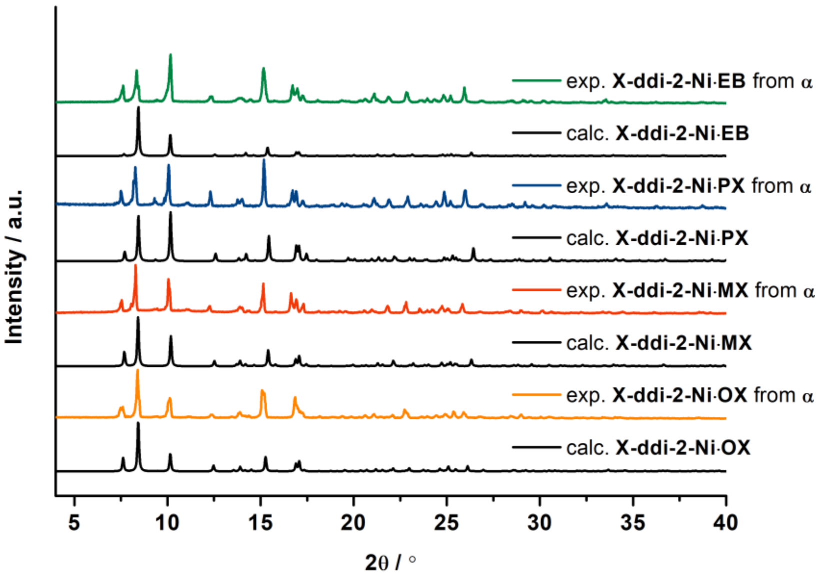

Figure S2. Calculated (black) and experimental PXRD patterns of **X-ddi-2-Ni-OX** (orange), **X-ddi-2-Ni-MX** (red), **X-ddi-2-Ni-PX** (blue) and **X-ddi-2-Ni-EB** (green) obtained from **X-ddi-2-Ni-α**.

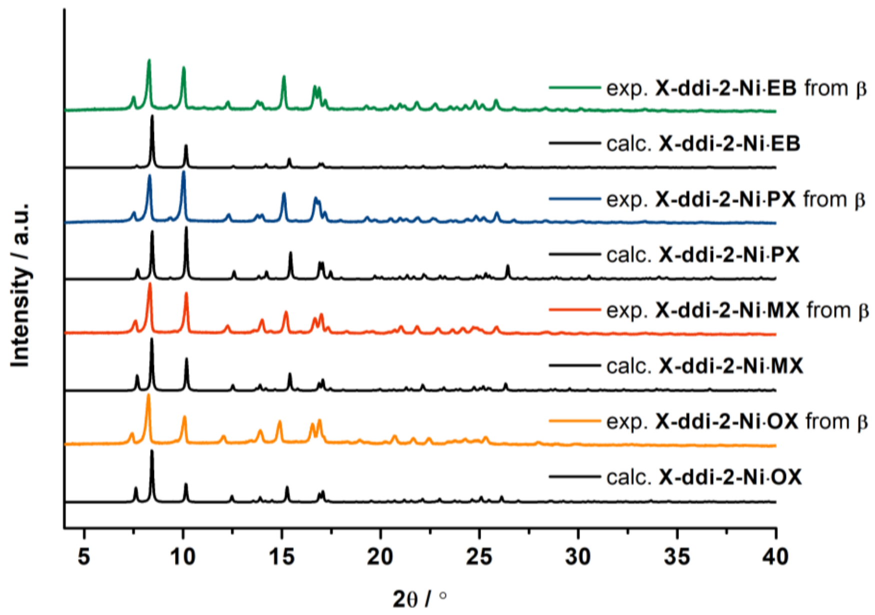

Figure S3. Calculated (black) and experimental PXRD patterns of **X-ddi-2-Ni-OX** (orange), **X-ddi-2-Ni-MX** (red), **X-ddi-2-Ni-PX** (blue) and **X-ddi-2-Ni-EB** (green) obtained from **X-ddi-2-Ni-β** upon soaking for one day.

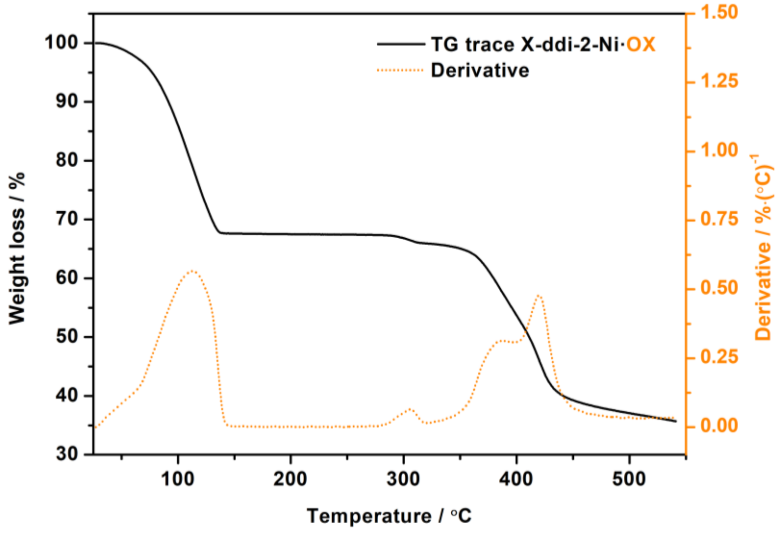

Figure S4. TG trace (black) and derivative (orange) of **X-ddi-2-Ni-OX**.

X-ddi-2-Ni-OX displayed a weight loss of 32.5% completed by *ca.* 140 °C, corresponding to two OX molecules per Ni unit (calc. 32.4%).

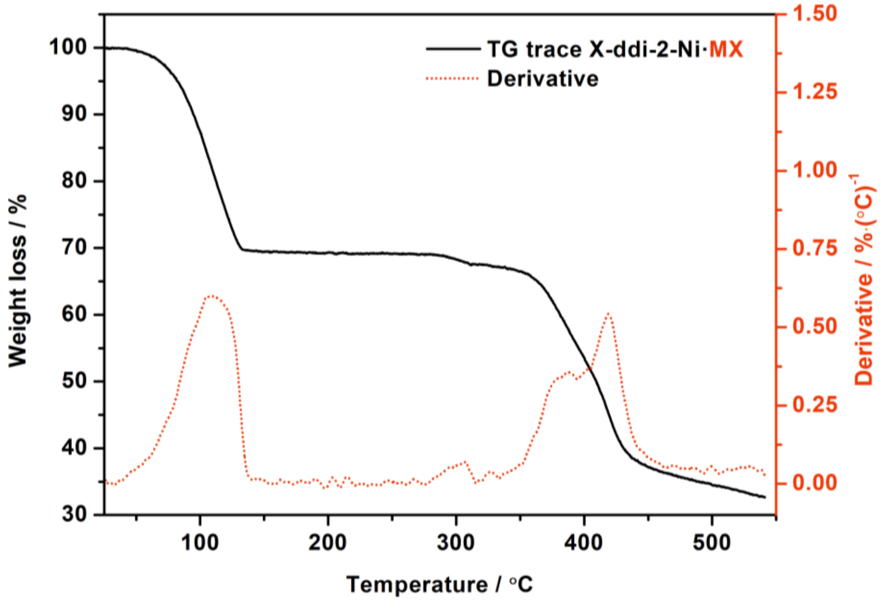

Figure S5. TG trace (black) and derivative (red) of **X-ddi-2-Ni-MX**.

X-ddi-2-Ni-MX displayed a weight loss of 30.6% completed by *ca.* 140 °C, corresponding to two MX molecules per Ni unit (calc. 32.4%).

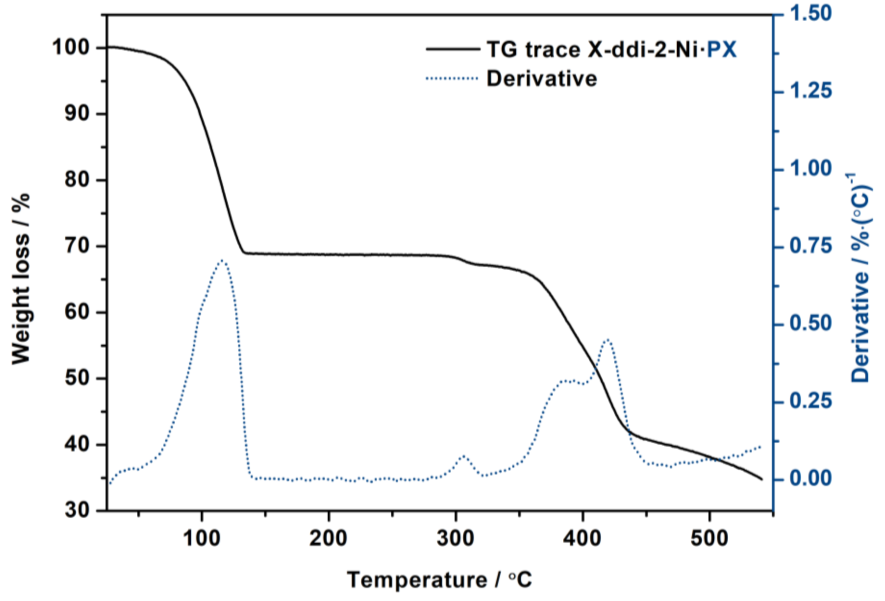

Figure S6. TG trace (black) and derivative (blue) of **X-ddi-2-Ni-PX**.

X-ddi-2-Ni-PX displayed a weight loss of 31.3% completed by *ca.* 140 °C, corresponding to two PX molecules per Ni unit (calc. 32.4%).

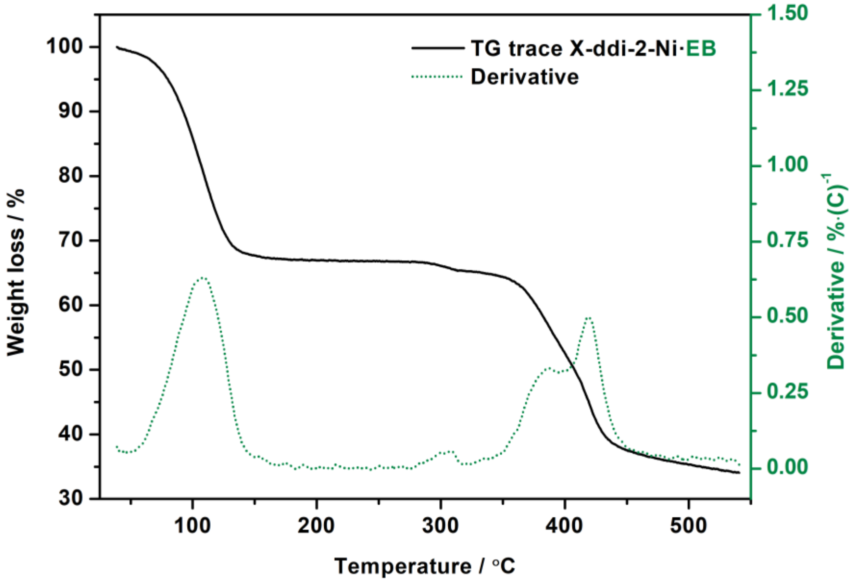

Figure S7. TG trace (black) and derivative (green) of **X-ddi-2-Ni-EB**.

X-ddi-2-Ni-EB displayed a weight loss of 32.8% completed by *ca.* 140 ˚C, corresponding to two EB moleculesper Ni unit (calc. 32.4%).

Table S2. Gate-opening pressure (P_{go}) calculations for **X-ddi-2-Ni-β** exposed to OX, MX, PX and EB at 298 K.

Guest	Uptake before P_{go} (Ub) [wt%]	Uptake after P_{go} (Ua) [wt%]	$\frac{U_a - U_b}{2}$ [wt%]	P_{go} corresponding to $\frac{U_a - U_b}{2}$ [P/P_0]
**OX**	0.2	48.9	24.4	14.5
**MX**	0.3	47.8	23.8	10.6
**PX**	0.3	45.9	22.8	10.4
**EB**	0.5	48.3	23.9	17.9

Table S3. Calculated (from TGA) and experimental (from vapor sorption isotherms) uptake of OX, MX, PX and EB in **X-ddi-2-Ni-β**.

Guest	TGA weight loss based on open phase [wt%]	Calculated uptake of closed phase [wt%]	Experimental uptake at 298 K [wt%]
**OX**	32.5	48.1	51.4
**MX**	30.6	44.1	49.9
**PX**	31.3	45.6	47.8
**EB**	32.8	48.8	50.5

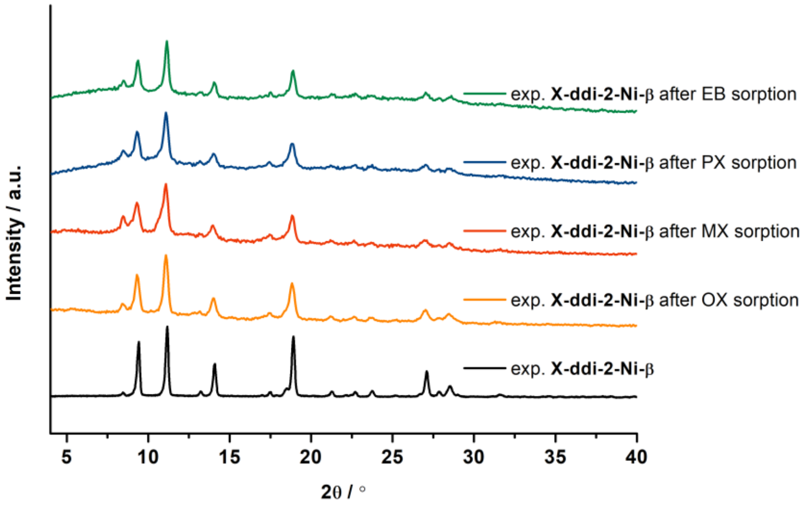

Figure S8. Experimental PXRD patterns of **X-ddi-2-Ni-β** (black) and PXRD patterns of **X-ddi-2-Ni** after desorption of OX (orange), MX (red), PX (blue) and EB (green) after multiple cycles (Figure S10).

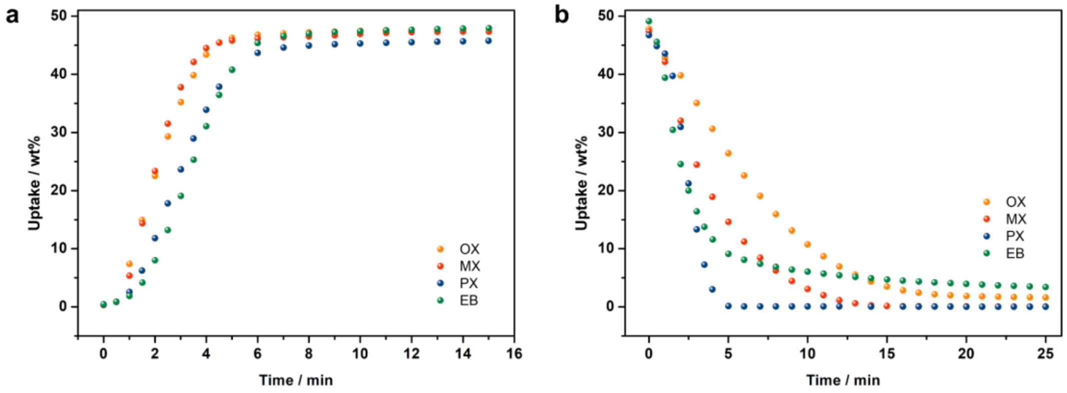

Figure S9. Kinetic data for the first cycle of adsorption (a) and desorption (b) of OX (orange), MX (red), PX (blue) and EB (green).

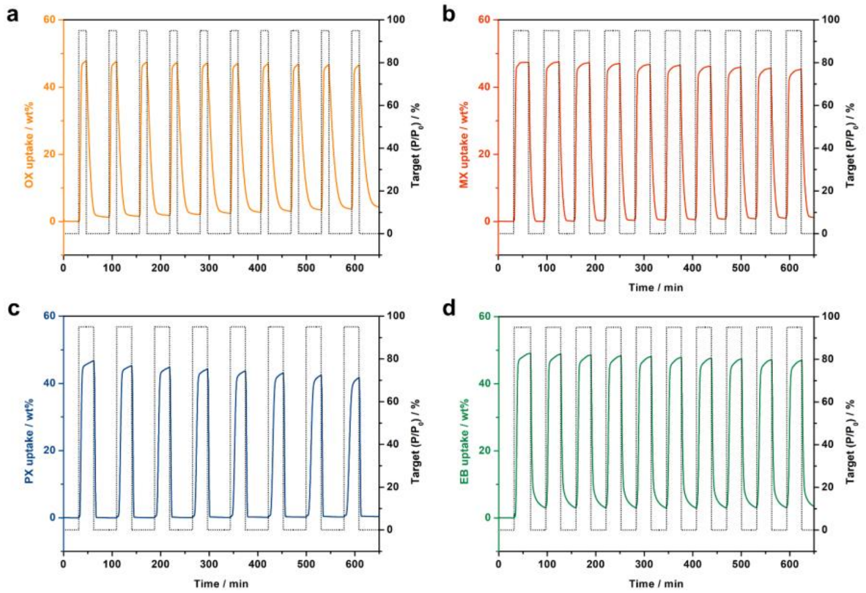

Figure S10. Cycling measurements over 8-10 adsorption-desorption cycles for OX (a), MX (b), PX (c) and EB (d).

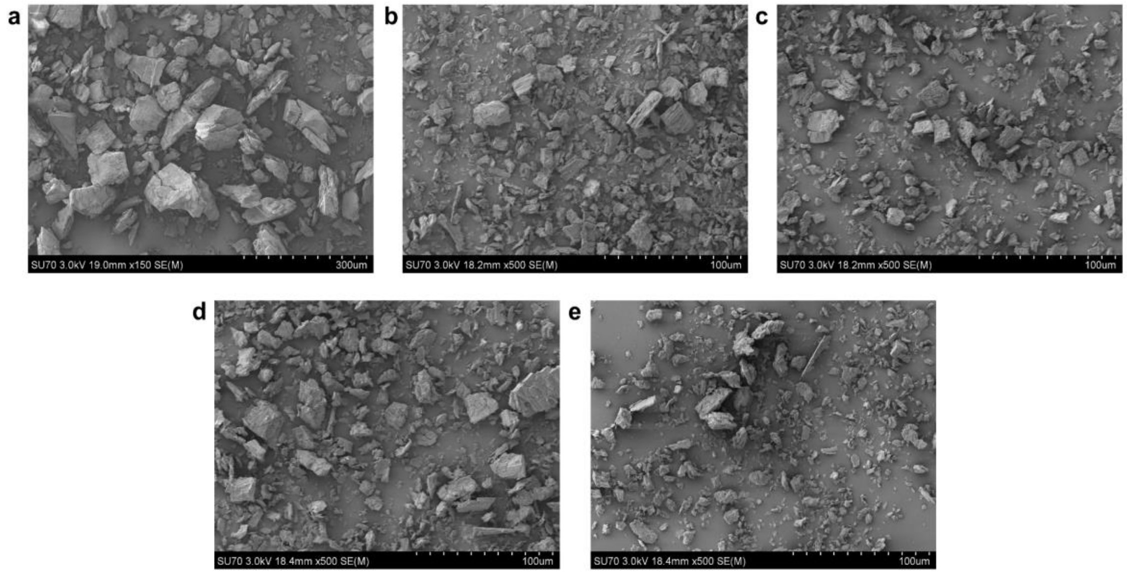

Figure S11. SEM images for **X-ddi-2-Ni-β** before C8 sorption (a) and after cycling sorption of OX (b), MX (c), PX (d) and EB (e).

Table S4. List of interactions in the experimental binding sites of OX, MX, PX and EB. Face-to-face interactions were measured between centroids constructed for the relevant aromatic rings. Edge-to-face interactions were measured between a centroid and the relevant carbon atom.

Binding site	Interaction	Distance in X-ddi-2-Ni-OX (Å)	Distance in X-ddi-2-Ni-MX (Å)	Distance in X-ddi-2-Ni-PX (Å)	Distance in X-ddi-2-Ni-EB (Å)
I	face-to-face π···π	3.818/3.532	3.683/3.574	3.635/3.602	3.767/3.698
	C-H···C van der Waals through $–CH_3$ (1) group	3.816	3.782	3.891/3.851	3.857/3.487
	C-H···C van der Waals through $–CH_3$ (2) group	3.469	3.392	3.549/3.808	-
	C-H···C van der Waals through $–CH_2$ group	-	-	-	3.676/3.588
II	face-to-face π–π	4.085	3.997	-	-
	C–H···π through $–CH_3$ group	3.867	4.099	-	-
	C–H···π through phenyl ring	4.159	4.047	-	-
III	C-H···C van der Waals through $–CH_3$ group	-	-	4.022	3.581/3.896*
	C-H···C van der Waals through phenyl ring	-	-	3.537/4.081	3.876/3.918*

Table S4 (continued). List of interactions in the experimental binding sites of OX, MX, PX and EB. Face-to-face interactions were measured between centroids constructed for the relevant aromatic rings. Edge-to-face interactions were measured between a centroid and the relevant carbon atom.

IV	C-H···π<br>through –CH_3 group	-	-	3.815	-
	C-H···π<br>through –CH_2 group	-	-	-	3.743*
	C-H···C van der Waals<br>through phenyl ring	-	-	3.440/3.556	3.695/<br>3.589/3.766*
	C-H···N<br>through –CH_3 group	-	-	3.542	-
	C-H···C van der Waals<br>through –CH_3 group	-	-	-	3.881/3.961*

*Calculated from computational binding sites

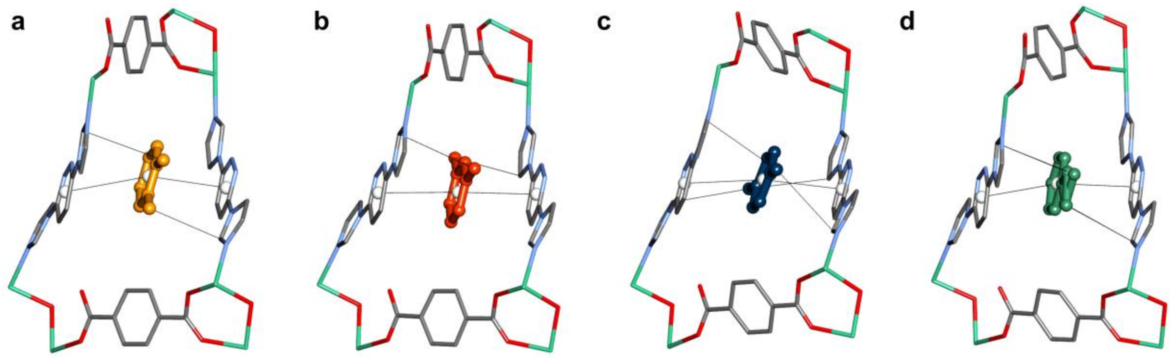

Figure S12. Host-guest interactions for OX (a), MX (b), PX (c) and EB (d) at site I. Full list of interactions is included in Table S4. Hydrogen atoms are omitted for clarity.

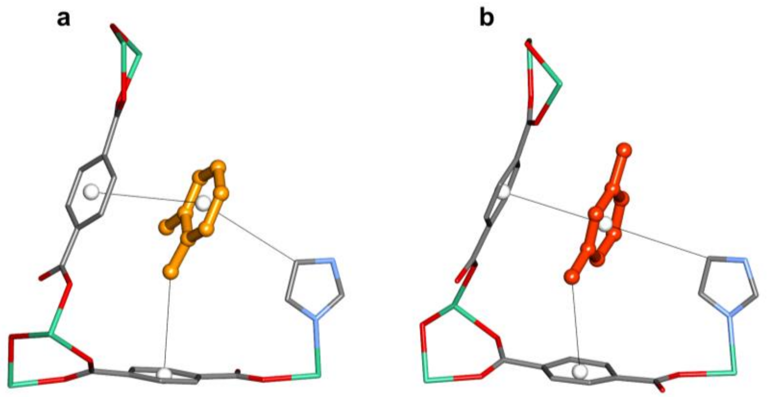

Figure S13. Host-guest interactions for OX (a) and MX (b) at site II. Full list of interactions is included in Table S4. Hydrogen atoms are omitted for clarity.

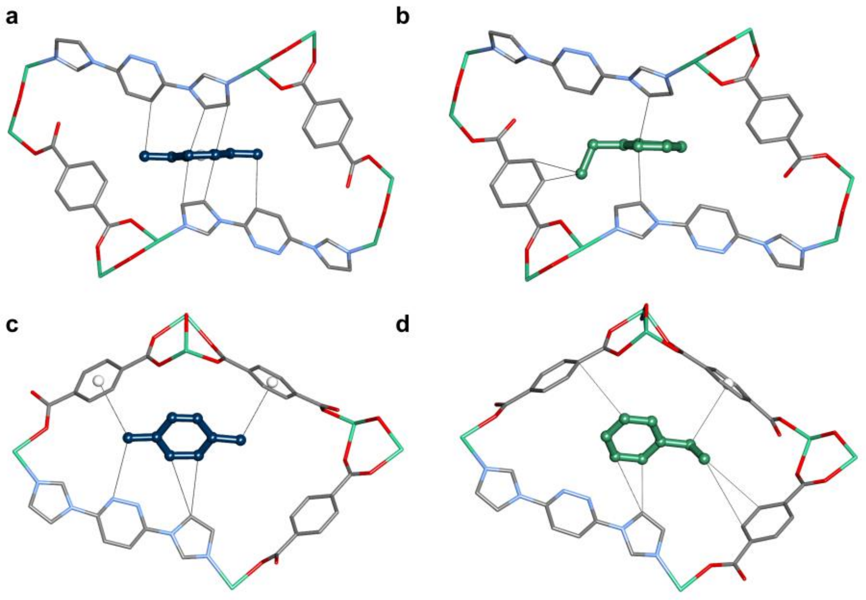

Figure S14. Host-guest interactions for PX (left) and EB (computational, right) at site III (a and b) and site IV (c and d). Full list of interactions is included in Table S4. Hydrogen atoms are omitted for clarity.

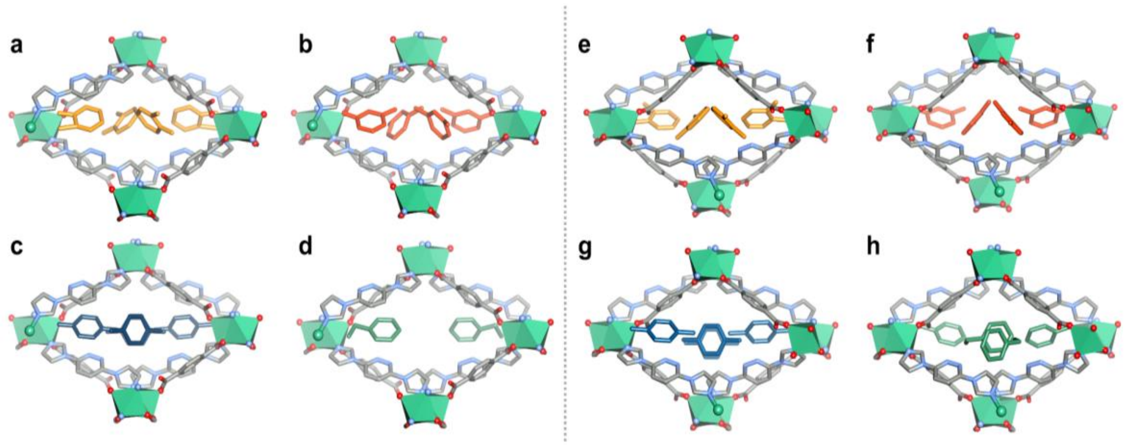

Figure S15. Comparison of experimental (left) and computational (right) binding sites for OX (a and e, respectively), MX (b and f, respectively), PX (c and g, respectively) and EB (d and h, respectively). Hydrogen atoms are omitted for clarity.

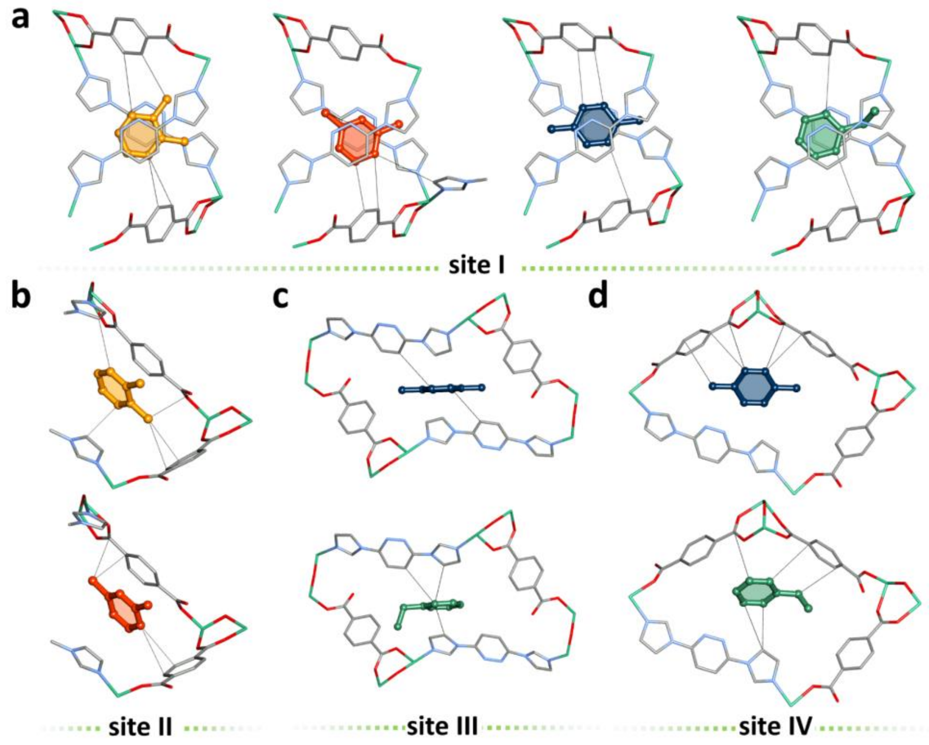

Figure S16. Computational binding sites of C8 isomers in **X-ddi-2-Ni**: site I (a), site II (b), site III (c) and site IV (d). Hydrogen atoms are omitted for clarity. Color codes: C, grey; N, blue; O, red; Ni, green. C8 guest molecules are shown in colours: OX, orange; MX, red; PX, blue; EB, green.

Table S5. List of interactions in the computed binding sites of OX, MX, PX and EB. Face-to-face interactions were measured between centroids constructed for the relevant aromatic rings. Edge-to-face interactions were measured between a centroid and the relevant carbon atom.

Binding site	Interaction	Distance in X-ddi-2-Ni-OX (Å)	Distance in X-ddi-2-Ni-MX (Å)	Distance in X-ddi-2-Ni-PX (Å)	Distance in X-ddi-2-Ni-EB (Å)
I	face-to-face π···π	3.689/3.699	3.714/3.622	3.711/3.558	3.673/3.627
	C-H···C van der Waals through $-CH_3$ (1) group	3.836	4.192	3.886/3.660	3.664/3.230
	C-H···C van der Waals through $-CH_3$ (2) group	3.455	3.685	3.722/3.804	-
	C-H···C van der Waals through $-CH_2$ group	-	-	-	3.811/3.782
II	face-to-face π–π	3.583	3.953	-	-
	C–H···π through $-CH_3$ group	3.553	-	-	-
	C–H···π through phenyl ring	3.765	3.688/3.854	-	-
	C-H···C van der Waals through phenyl ring	3.884	-	-	-
III	C-H···C van der Waals through $-CH_3$ group	-	-	4.229	3.581/3.896
	C-H···C van der Waals through phenyl ring	-	-	4.047/4.054	3.876/3.918

Table S5 (continued). List of interactions in the computed binding sites of OX, MX, PX and EB. Face-to-face interactions were measured between centroids constructed for the relevant aromatic rings. Edge-to-face interactions were measured between a centroid and the relevant carbon atom.

IV	C-H···π<br>through $-CH_3$ group	-	-	3.663/3.664	-
	C-H···π<br>through $-CH_2$ group	-	-	-	3.743
	C-H···C van der Waals<br>through phenyl ring	-	-	3.360/3.594	3.695/<br>3.589/3.766
	C-H···N<br>through $-CH_3$ group	-	-	3.575	-
	C-H···C van der Waals<br>through $-CH_3$ group	-	-	-	3.881/3.961

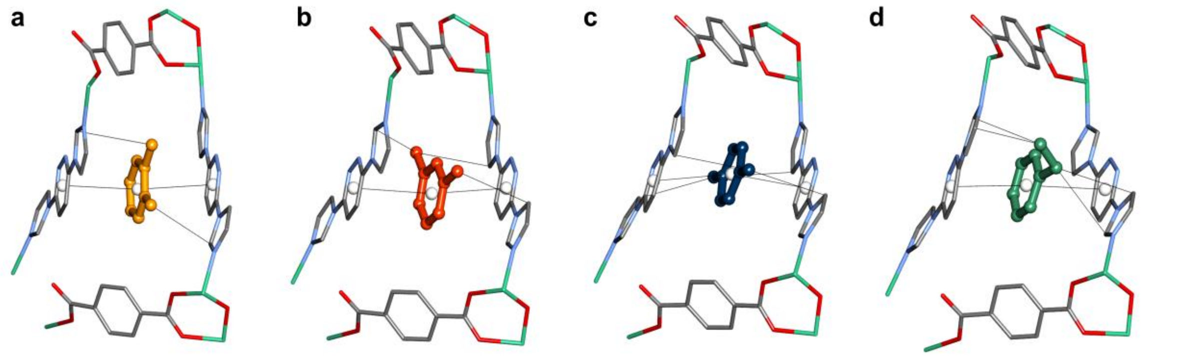

Figure S17. Computed host-guest interactions for OX (a), MX (b), PX (c) and EB (d) at site I. Full list of interactions is included in Table S5. Hydrogen atoms are omitted for clarity.

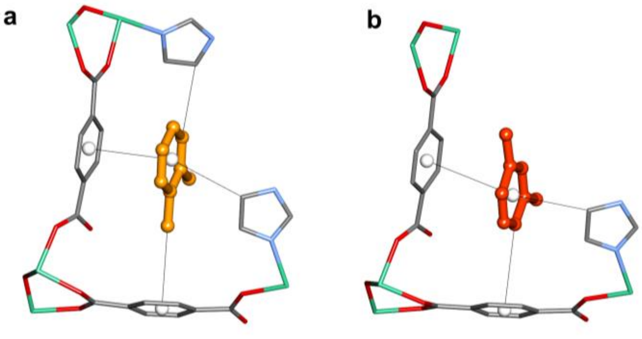

Figure S18. Computed host-guest interactions for OX (a) and MX (b) at site II. Full list of interactions is included in Table S5. Hydrogen atoms are omitted for clarity.

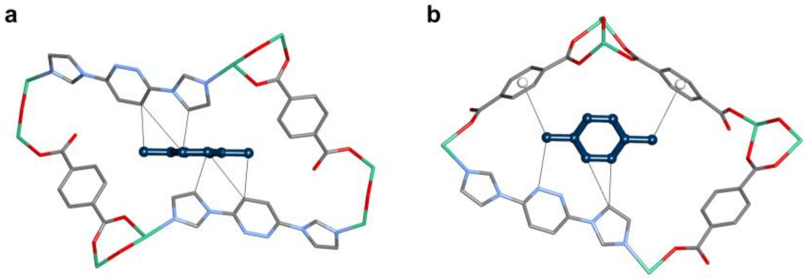

Figure S19. Computed host-guest interactions for PX at site III (a) and site IV (b). Full list of interactions is included in Table S5. Hydrogen atoms are omitted for clarity.

Table S6. Selectivity values for separation of C8 isomers using **X-ddi-2-Ni** measured by NMR.

Selectivity	Measured from NMR
$S_{OX/MX}$	0.97
$S_{OX/PX}$	0.90
$S_{OX/EB}$	1.06
$S_{MX/PX}$	1.01
$S_{MX/EB}$	1.06
$S_{PX/EB}$	1.15

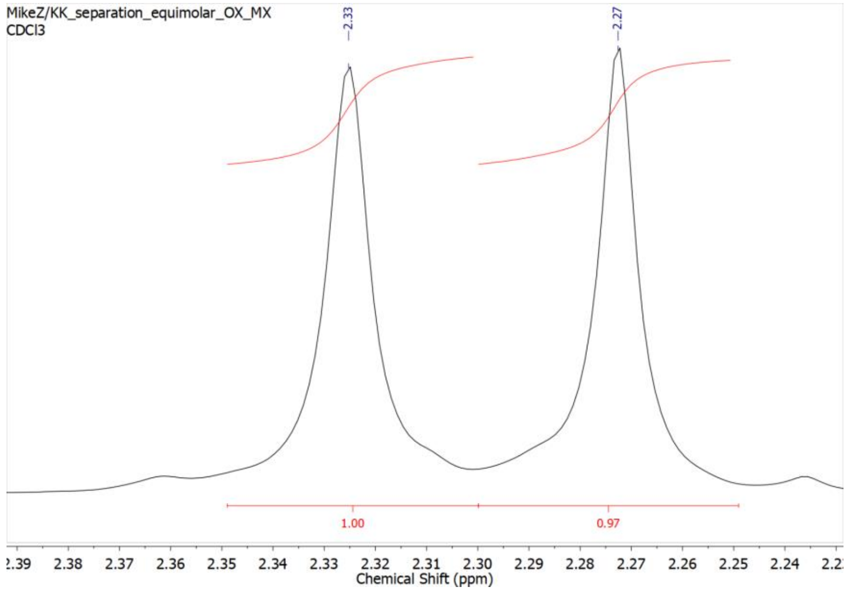

Figure S20. NMR spectrum of supernatant of **X-ddi-2-Ni** soaked in binary equimolar mixture of OX/MX diffused in $CDCl_3$.

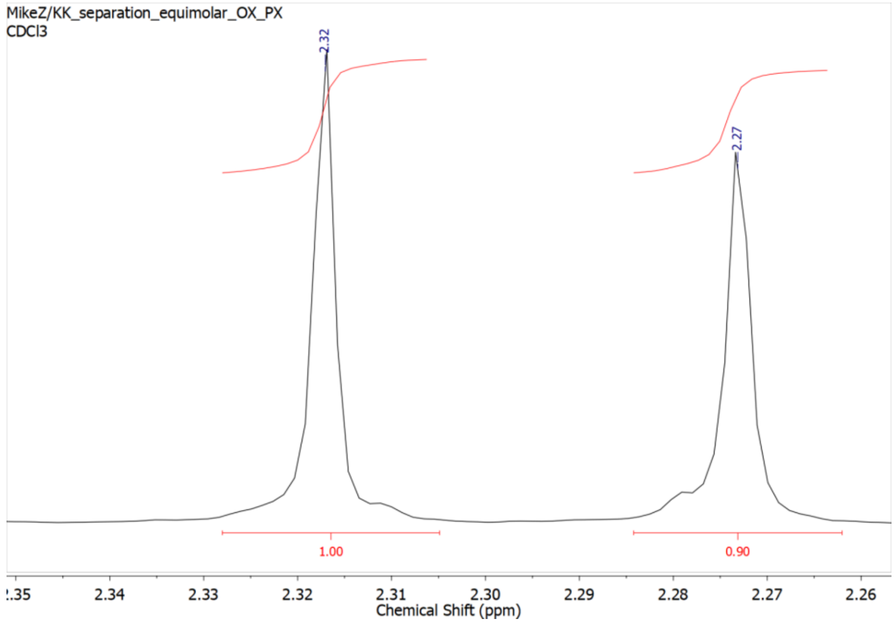

Figure S21. NMR spectrum of supernatant of **X-ddi-2-Ni** soaked in binary equimolar mixture of OX/PX diffused in $CDCl_3$.

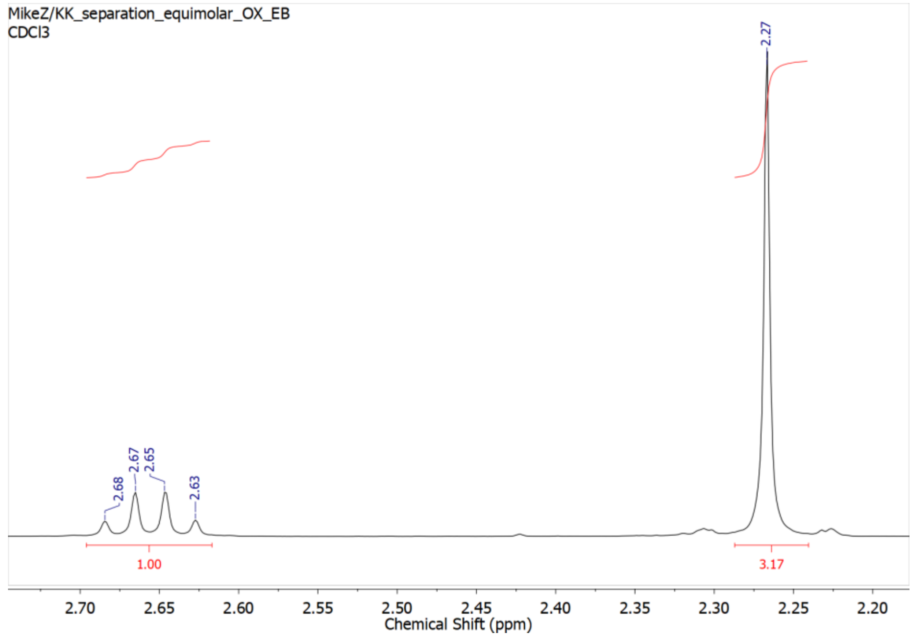

Figure S22. NMR spectrum of supernatant of **X-ddi-2-Ni** soaked in binary equimolar mixture of OX/EB diffused in $CDCl_3$.

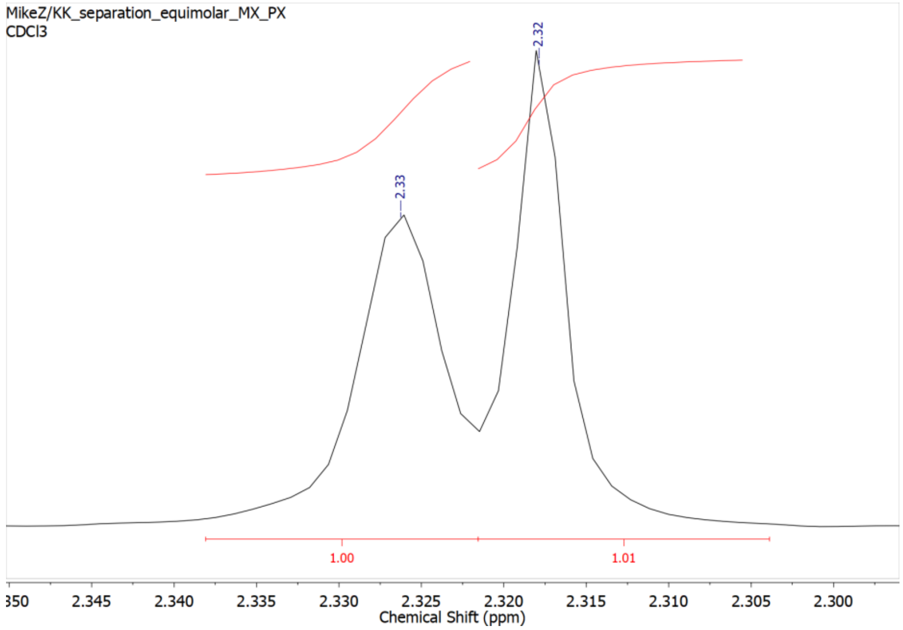

Figure S23. NMR spectrum of supernatant of **X-ddi-2-Ni** soaked in binary equimolar mixture of MX/PX in diffused in $CDCl_3$.

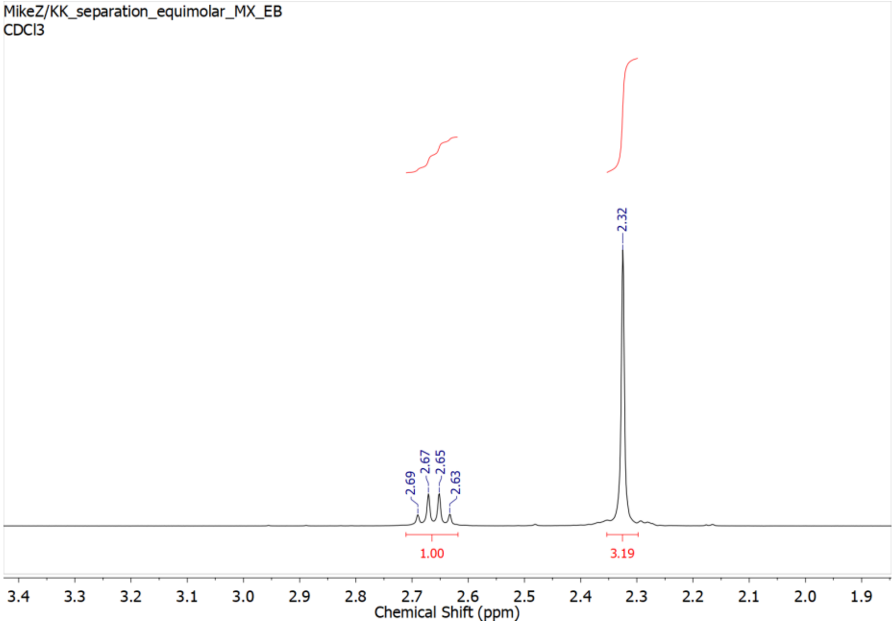

Figure S24. NMR spectrum of supernatant of X-ddi-2-Ni soaked in binary equimolar mixture of MX/EB diffused in CDCl3.

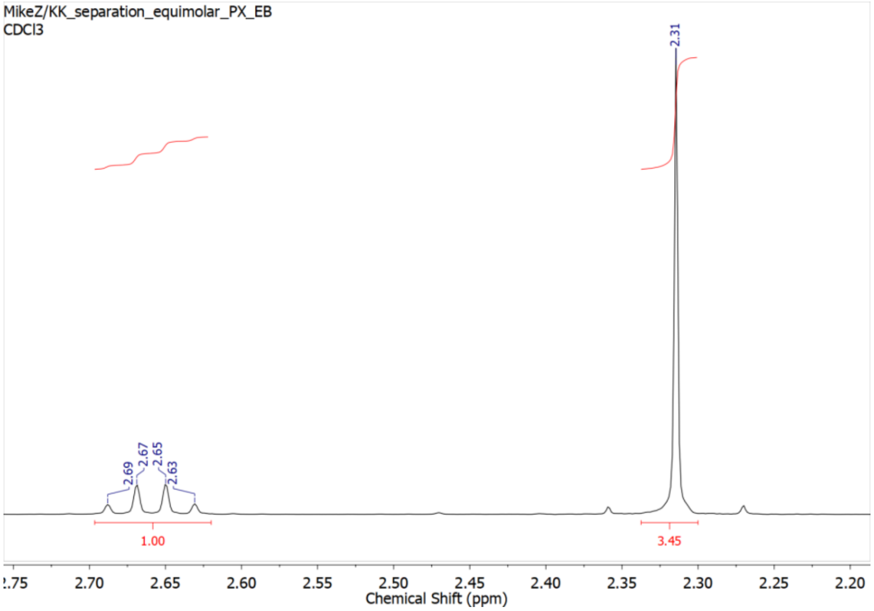

Figure S25. NMR spectrum of supernatant of **X-ddi-2-Ni** soaked in binary equimolar mixture of PX/EB diffused in $CDCl_3$.

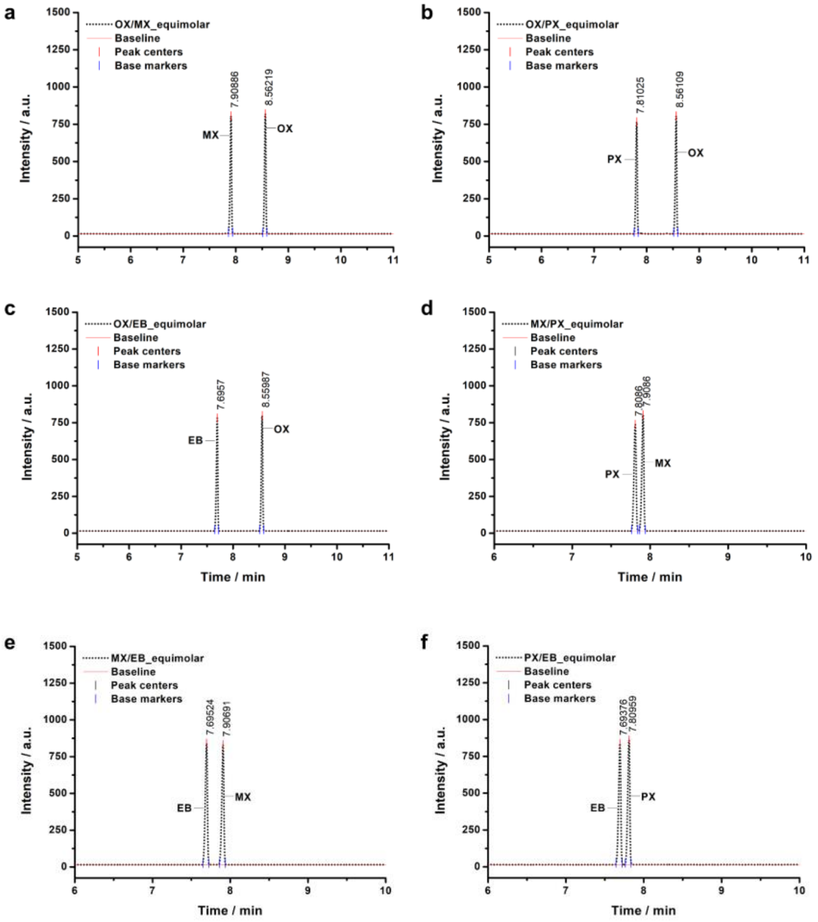

Figure S26. GC chromatograms of equimolar binary mixtures of OX/MX (a), OX/PX (b), OX/EB (c), MX/PX (d), MX/EB (e) and PX/EB (f).

Table S7. Mole fractions of components in the adsorbed phase calculated by GC experiments. Chromatogram analysis is shown in Figure S31.

Solution	Areas in solutions		Peak position (min)	Mole fractions	
**OX/MX_equimolar**	OX	25.96496	8.5622	X_{OX}	0.5063
	MX	25.31690	7.9089	X_{MX}	0.4937
**OX/PX_equimolar**	OX	24.86984	8.5611	X_{OX}	0.5071
	PX	24.17039	7.8103	X_{PX}	0.4929
**OX/EB_equimolar**	OX	24.95909	8.5599	X_{OX}	0.5033
	EB	24.63304	7.6957	X_{EB}	0.4967
**MX/PX_equimolar**	MX	24.49741	7.9086	X_{MX}	0.5038
	PX	24.12480	7.8086	X_{PX}	0.4962
**MX/EB_equimolar**	MX	25.88176	7.9069	X_{MX}	0.4944
	EB	26.47056	7.6952	X_{EB}	0.5056
**PX/EB_equimolar**	PX	26.77484	7.8096	X_{PX}	0.4937
	EB	27.45638	7.6938	X_{EB}	0.5063

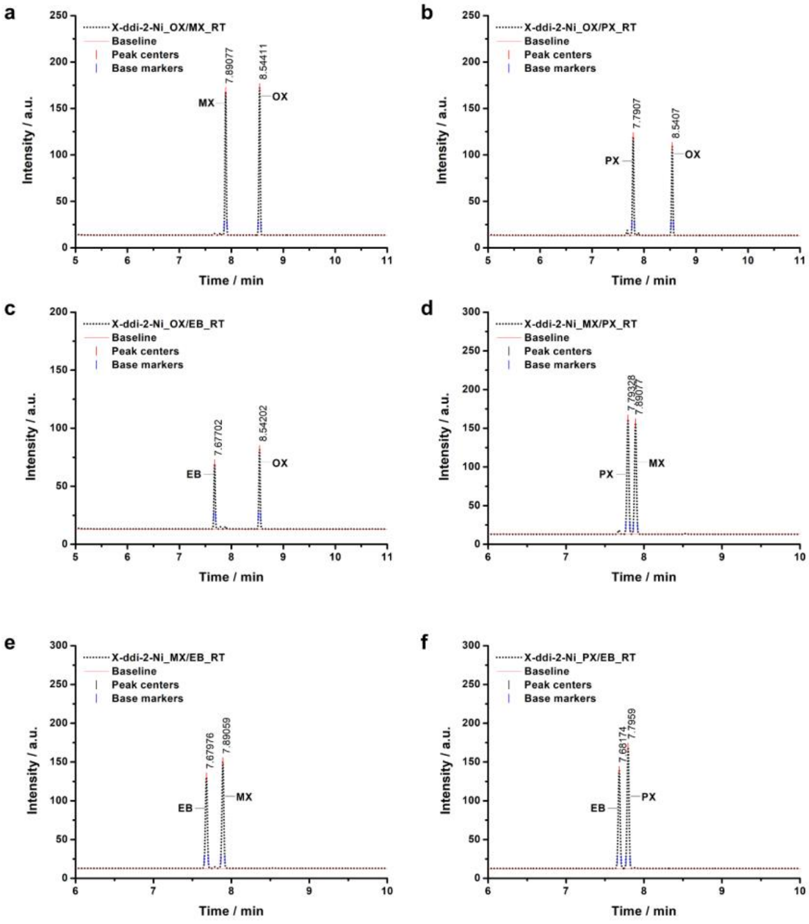

Figure S27. GC chromatograms of supernatant of **X-ddi-2-Ni** soaked in binary equimolar mixtures of OX/MX (a), OX/PX (b), OX/EB (c), MX/PX (d), MX/EB (e) and PX/EB (f) at room temperature (RT).

Table S8. Mole fractions of components in the liquid phase calculated by GC experiments after soaking **X-dd-2-Ni** in binary equimolar C8 mixtures at room temperature (RT). Chromatogram analysis is shown in Figure S32.

Sample	Areas in sample		Peak position (min)	Mole fractions	
**X-ddi-2-Ni_OX/MX_RT**	OX	4.69418	8.5441	Y_{OX}	0.5091
	MX	4.52571	7.8908	Y_{MX}	0.4909
**X-ddi-2-Ni_OX/PX_RT**	OX	2.72502	8.5407	Y_{OX}	0.4702
	PX	3.07091	7.7907	Y_{PX}	0.5298
**X-ddi-2-Ni_OX/EB_RT**	OX	1.94872	8.5420	Y_{OX}	0.5547
	EB	1.56423	7.6770	Y_{EB}	0.4453
**X-ddi-2-Ni_MX/PX_RT**	MX	4.14741	7.8908	Y_{MX}	0.4852
	PX	4.40060	7.7933	Y_{PX}	0.5148
**X-ddi-2-Ni_MX/EB_RT**	MX	3.90748	7.8906	Y_{MX}	0.5326
	EB	3.42858	7.6798	Y_{EB}	0.4674
**X-ddi-2-Ni_PX/EB_RT**	PX	4.52338	7.7959	Y_{PX}	0.5516
	EB	3.67760	7.6817	Y_{EB}	0.4484

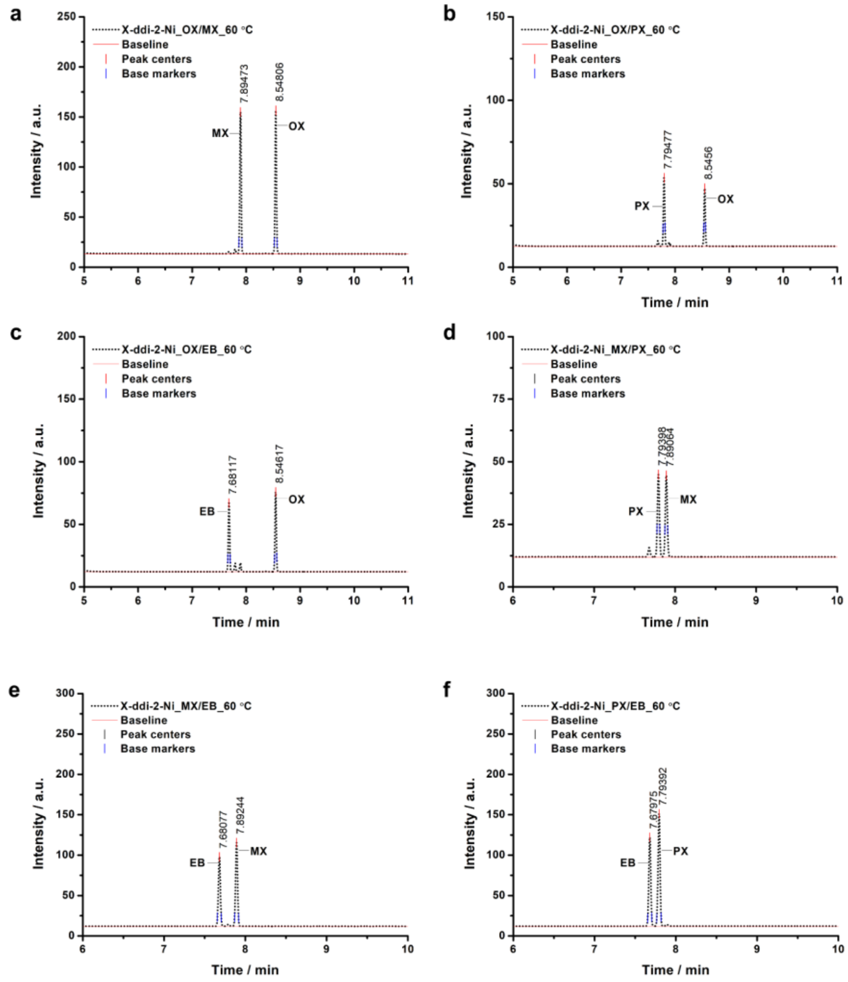

Figure S28. GC chromatograms of supernatant of **X-ddi-2-Ni** soaked in binary equimolar mixtures of OX/MX (a), OX/PX (b), OX/EB (c), MX/PX (d), MX/EB (e) and PX/EB (f) at 60 °C.

Table S9. Mole fractions of components in the liquid phase calculated by GC experiments after soaking **X-dd-2-Ni** in binary equimolar C8 mixtures at 60 ˚C. Chromatogram analysis is shown in Figure S33.

Sample	Areas in sample		Peak position (min)	Molar fractions	
**X-ddi-2-Ni_OX/MX_60˚C**	OX	4.16809	8.5481	Y_{OX}	0.5024
	MX	4.12896	7.8947	Y_{MX}	0.4976
**X-ddi-2-Ni_OX/PX_60˚C**	OX	0.89054	8.5456	Y_{OX}	0.4456
	PX	1.10791	7.7948	Y_{PX}	0.5544
**X-ddi-2-Ni_OX/EB_60˚C**	OX	1.77512	8.5462	Y_{OX}	0.5359
	EB	1.53736	7.6812	Y_{EB}	0.4641
**X-ddi-2-Ni_MX/PX_60˚C**	MX	0.83607	7.8906	Y_{MX}	0.5018
	PX	0.83007	7.7940	Y_{PX}	0.4982
**X-ddi-2-Ni_MX/EB_60˚C**	MX	2.93186	7.8924	Y_{MX}	0.5473
	EB	2.42484	7.6808	Y_{EB}	0.4527
**X-ddi-2-Ni_PX/EB_60˚C**	PX	3.98156	7.7939	Y_{PX}	0.5600
	EB	3.12793	7.6798	Y_{EB}	0.4400

Table S10. Selectivity values for separation of C8 isomers using **X-ddi-2-Ni**, measured by GC.

Sample (RT)	Selectivity		$\lvert S-1 \rvert$	Sample (60C)	Selectivity		$\lvert S-1 \rvert$
**X-ddi-2-Ni _OX/MX_RT**	$S_{OX/MX}$	1.0113	0.0113	**X-ddi-2-Ni _OX/MX_60C**	$S_{OX/MX}$	0.9845	0.0155
**X-ddi-2-Ni _OX/PX_RT**	$S_{OX/PX}$	0.8626	0.1374	**X-ddi-2-Ni _OX/PX_60C**	$S_{OX/PX}$	0.7812	0.2188
**X-ddi-2-Ni _OX/EB_RT**	$S_{OX/EB}$	1.2293	0.2293	**X-ddi-2-Ni _OX/EB_60C**	$S_{OX/EB}$	1.1396	0.1396
**X-ddi-2-Ni _MX/PX_RT**	$S_{MX/PX}$	0.9283	0.0717	**X-ddi-2-Ni _MX/PX_60C**	$S_{MX/PX}$	0.9920	0.0080
**X-ddi-2-Ni _MX/EB_RT**	$S_{MX/EB}$	1.1653	0.1653	**X-ddi-2-Ni _MX/EB_60C**	$S_{MX/EB}$	1.2364	0.2364
**X-ddi-2-Ni_ PX/EB_RT**	$S_{PX/EB}$	1.2615	0.2615	**X-ddi-2-Ni _PX/EB_60C**	$S_{PX/EB}$	1.3052	0.3052

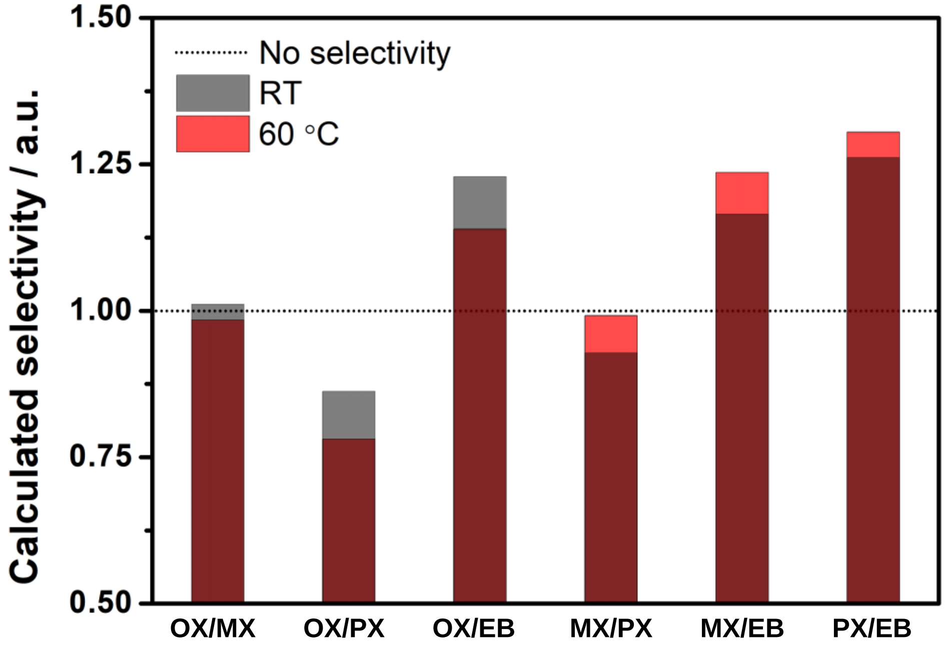

Figure S29. Comparison of selectivity values for separation of C8 isomers using **X-ddi-2-Ni** measured by GC at room temperature (RT, grey bars) and 60 °C (red bars). Black dotted line at selectivity value of 1 corresponds to no isomer recognition.

Table S11. Uptakes of C_8 isomers and gate-opening pressures (P_{go}) of reported flexible sorbents for the separation of C8 aromatics.

Material	OX uptake (wt%)	P_{go} OX (% P/P_0)	MX uptake (wt%)	P_{go} MX (% P/P_0)	PX uptake (wt%)	P_{go} PX (% P/P_0)	EB uptake (wt%)	P_{go} EB (% P/P_0)	Ref.
sql-1-Co-NCS	85.2	25	82.8	50	84.8	30	37.2	70	11
X-ddi-2-Ni	51.4	14.5	49.9	10.6	47.8	10.4	50.5	17.9	this work
Zn(o-phen)(2,6-ndc)	35.3	5	43.8	3	41.1	3	0	No	12
ZUL-C3	36.2	<2.3	35.6	<2.3	34.7	2.3	34.5	<2.3	13
MIL-53(Fe)	35	4	42.4	10	33.9	10	26.5	20	14
MIL-53(Al)	42	2	37.3	2	36.1	2	27.7	3	
MIL-53(Ga)	37.1	2	32.9	4	39.2	1	23.3	4	
MIL-53(Cr)	42.4	3	27.6	3	42.4	1	25.4	3	
HIAM-203	20	<5*	15.9	<5*	19.9	5	N/A	N/A	15
[Mn(dhbq)(H_2O)$_2$]	2.3	27	15.9	<5	18.5	<5	N/A	N/A	16
CAU-13	17	N/A	15	N/A	14	N/A	N/A	N/A	17
PCP-1	16.3	5	14.8	7	14.8	7	N/A	N/A	18
sql-1,3-Co-NCS	1	No	13	55	19	40	2.5	No	19
sql-4,5-Zn	5	No	3.3	No	20.7	35	15.9	60	20
JUC-77	0.9	No	2.3	No	33	40	N/A	N/A	21
DynaMOF-100	0.53	No	2.12	No	31.8	10	3.82	No	22
SAMM-3-Cu-OTf	42	90	0	No	0	No	0	No	23
[Ce(HTCPB)]	N/A	N/A	12.7	N/A	11.7	N/A	N/A	N/A	24
MCF-50	N/A	N/A	N/A	N/A	N/A	N/A	N/A	N/A	25
Ni(NCS)$_2$(ppp)$_4$	29	N/A	27	N/A	38	N/A	N/A	N/A	26

*At 333 K. N/A: the isotherm is not reported. No: P_{go} is not observed in the reported isotherm.

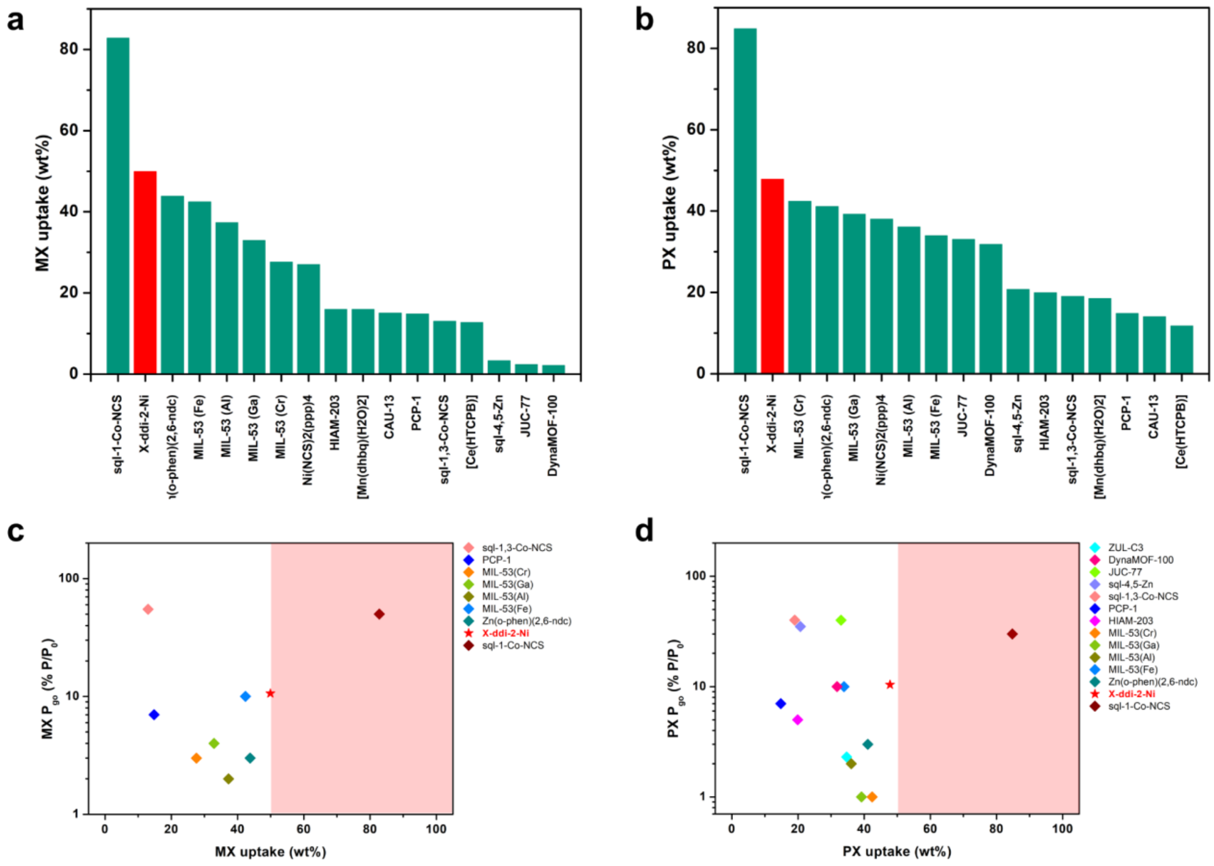

Figure S30. Comparison of uptake of (a) MX and (b) PX for **X-ddi-2-Ni** (red) and other flexible sorbents (green). P_{go} values plotted against uptake of (c) MX and (d) PX for **X-ddi-2-Ni** and other flexible sorbents. Red shaded area represents the area of high adsorptive capacity (≥ 50 wt%).

Table S12. Uptakes of C_8 isomers and pore size of reported rigid sorbents for the separation of C8 aromatics.

Platform	Pore Dimensions (atom to atom, Å)	OX uptake (wt%)	MX uptake (wt%)	PX uptake (wt%)	EB uptake (wt%)	Ref.
MIL-47*	11 × 11	35	28	37	16	27
MOF-48(V)	10 × 10	27.6	27.6	27.6	27.6	28
MIL-101(Cr)	13 × 14	123	123	133	N/A	29
MIL-125-NH_2	7 × 9	10	11	14.5	N/A	30
MIL-140B	5 × 7	12.7	12.7	12.7	12.7	28
MOF-5	11 × 11	13	14.5	13	10	31
MOF-monoclinic	N/A	4.2	4.2	12.5	4.2	
Zn(BDC)(Dabco)$_{0.5}$	7 × 8	25	27	23	27	32
ZIF-8	6 × 8	1.6	3.2	15.9	N/A	33
UiO-66	12 × 12	42.4	42.4	42.4	N/A	34
HKUST-1	10 × 10	29.7	25.4	29.7	N/A	35
CPO-27-Ni	15 × 15	20.1	22.3	21.2	N/A	
Co_2(dobdc)	13 × 16	38.2	36.0	35.0	35.0	36
Co_2(m-dobdc)	15 × 15	36.0	35.5	33.2	35.5	
MAF-X8	11 × 12	1.6	11.1	22.3	0	37
Zn-MOF	9 × 9	0	0.11	0.42	N/A	38
MFM-300(In)	13 × 13	29.8	29.2	30.5	N/A	39
MFM-300(V)		38.9	41.2	36.6	N/A	
MFM-300(Al)		4.9	4.8	8.7	N/A	
SIFSIX-1-Cu	10 × 10	13	11.2	7.9	N/A	40
EtP5	N/A	0.2	1.1	8.9	--	41
EtP6		9.4	9.1	9.9		
K/ZSM-5		2	1.2	8.3	7.5	42
H/ZSM-5		3.05	1.24	14.22	6.03	
Li/ZSM-5		4.3	2.5	11	5.5	
Na/ZSM-5		3	2.5	9	5.5	
KaX		4.2	1.8	10.1	3.2	43
BaX		4.9	2.02	10.34	3.15	44

*No gate-opening observed for C8 isomers so therefore included in the rigid materials table

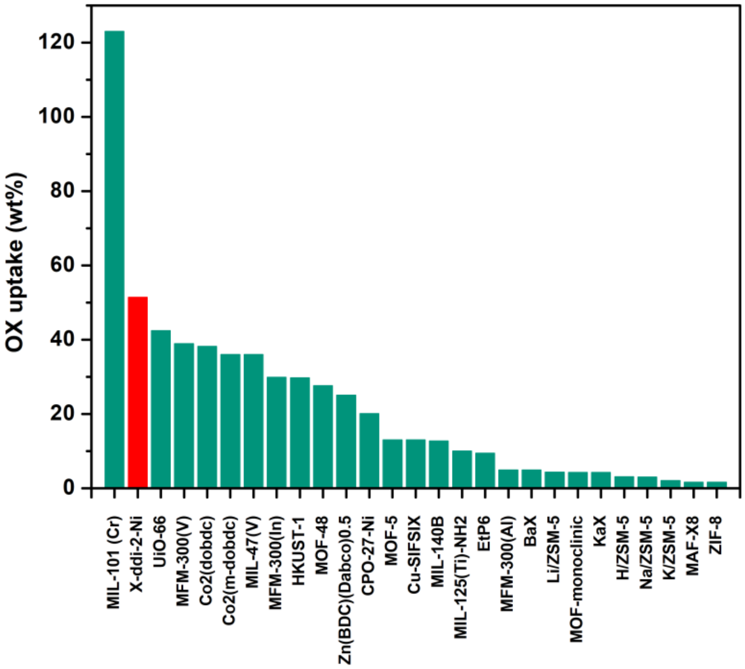

Figure S31. Comparison of OX uptake for **X-ddi-2-Ni** (red) and rigid sorbents (green) for C8 sorption and separation.

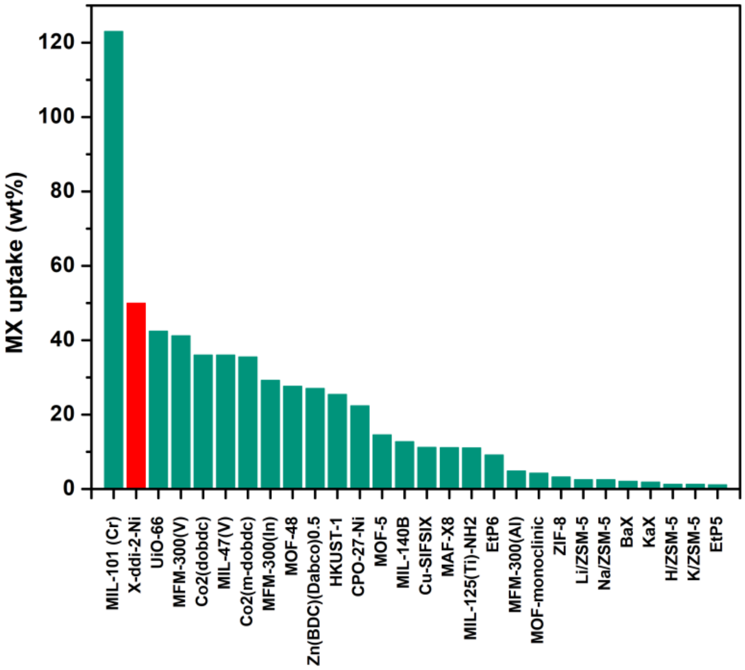

Figure S32. Comparison of MX uptake for **X-ddi-2-Ni** (red) and rigid sorbents (green) for C8 sorption and separation.

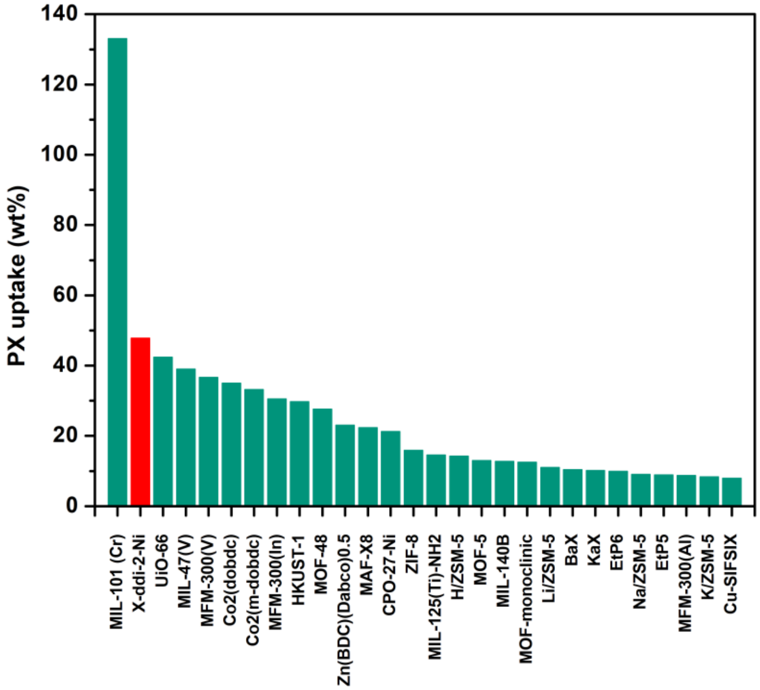

Figure S33. Comparison of PX uptake for **X-ddi-2-Ni** (red) and rigid sorbents (green) for C8 sorption and separation.

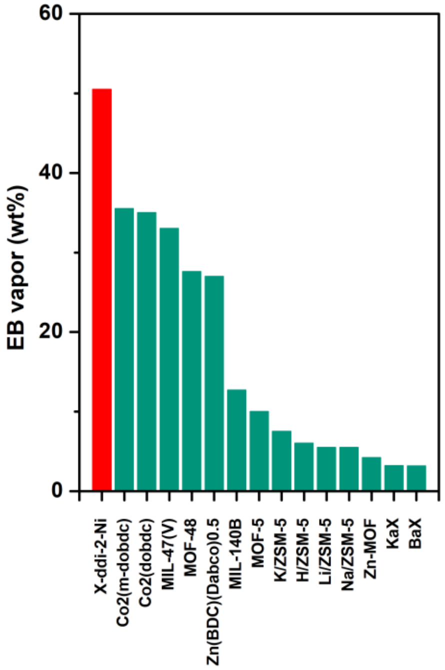

Figure S34. Comparison of EB uptake for **X-ddi-2-Ni** (red) and rigid sorbents (green) for C8 sorption and separation.

S3. References

1. Koupepidou, K.; Nikolayenko, V. I.; Sensharma, D.; Bezrukov, A. A.; Shivanna, M.; Castell, D. C.; Wang, S.-Q.; Kumar, N.; Otake, K.-i.; Kitagawa, S.; Zaworotko, M. J., Control over Phase Transformations in a Family of Flexible Double Diamondoid Coordination Networks through Linker Ligand Substitution. *Chemistry of Materials* **2023,** *35* (9), 3660-3670.

2. Francart, T.; van Wieringen, A.; Wouters, J., APEX 3: a multi-purpose test platform for auditory psychophysical experiments. *J Neurosci Methods* **2008,** *172* (2), 283-93.

3. Sheldrick, G., sadabs, Version 2008/1, Bruker AXS. *Inc.: Madison, WI* **2008.**

4. Dolomanov, O. V.; Bourhis, L. J.; Gildea, R. J.; Howard, J. A. K.; Puschmann, H., OLEX2: a complete structure solution, refinement and analysis program. *Journal of Applied Crystallography* **2009,** *42* (2), 339-341.

5. Spek, A., PLATON SQUEEZE: a tool for the calculation of the disordered solvent contribution to the calculated structure factors. *Acta Crystallographica Section C* **2015,** *71* (1), 9-18.

6. Blöchl, P. E., Projector augmented-wave method. *Physical Review B* **1994,** *50* (24), 17953-17979.

7. Kresse, G.; Furthmüller, J., Efficiency of ab-initio total energy calculations for metals and semiconductors using a plane-wave basis set. *Computational Materials Science* **1996,** *6* (1), 15-50.

8. Kresse, G.; Furthmüller, J., Efficient iterative schemes for ab initio total-energy calculations using a plane-wave basis set. *Physical Review B* **1996,** *54* (16), 11169-11186.

9. Wellendorff, J.; Lundgaard, K. T.; Møgelhøj, A.; Petzold, V.; Landis, D. D.; Nørskov, J. K.; Bligaard, T.; Jacobsen, K. W., Density functionals for surface science: Exchange-correlation model development with Bayesian error estimation. *Physical Review B* **2012,** *85* (23), 235149.

10. Monkhorst, H. J.; Pack, J. D., Special points for Brillouin-zone integrations. *Physical Review B* **1976,** *13* (12), 5188-5192.

11. Wang, S.-Q.; Mukherjee, S.; Patyk-Kaźmierczak, E.; Darwish, S.; Bajpai, A.; Yang, Q.-Y.; Zaworotko, M. J., Highly Selective, High-Capacity Separation of o-Xylene from C8 Aromatics by a Switching Adsorbent Layered Material. *Angewandte Chemie International Edition* **2019,** *58* (20), 6630-6634.

12. Laha, S.; Haldar, R.; Dwarkanath, N.; Bonakala, S.; Sharma, A.; Hazra, A.; Balasubramanian, S.; Maji, T. K., A Dynamic Chemical Clip in Supramolecular Framework for Sorting Alkylaromatic Isomers using Thermodynamic and Kinetic Preferences. *Angewandte Chemie International Edition* **2021,** *60* (36), 19921-19927.

13. Zhou, J.; Ke, T.; Song, Y.; Cai, H.; Wang, Z. a.; Chen, L.; Xu, Q.; Zhang, Z.; Bao, Z.; Ren, Q.; Yang, Q., Highly Efficient Separation of C8 Aromatic Isomers by Rationally Designed Nonaromatic Metal–Organic Frameworks. *Journal of the American Chemical Society* **2022,** *144* (46), 21417-21424.

14. Agrawal, M.; Bhattacharyya, S.; Huang, Y.; Jayachandrababu, K. C.; Murdock, C. R.; Bentley, J. A.; Rivas-Cardona, A.; Mertens, M. M.; Walton, K. S.; Sholl, D. S.; Nair, S., Liquid-Phase

Multicomponent Adsorption and Separation of Xylene Mixtures by Flexible MIL-53 Adsorbents. *The Journal of Physical Chemistry C* **2018,** *122* (1), 386-397.
15. Yu, L.; Zhang, J.; Ullah, S.; Yao, J.; Luo, H.; Huang, J.; Xia, Q.; Thonhauser, T.; Li, J.; Wang, H., Separating Xylene Isomers with a Calcium Metal-Organic Framework. *Angewandte Chemie International Edition* **2023,** *62* (41), e202310672.
16. Li, L.; Guo, L.; Olson, D. H.; Xian, S.; Zhang, Z.; Yang, Q.; Wu, K.; Yang, Y.; Bao, Z.; Ren, Q.; Li, J., Discrimination of xylene isomers in a stacked coordination polymer. *Science* **2022,** *377* (6603), 335-339.
17. Niekiel, F.; Lannoeye, J.; Reinsch, H.; Munn, A. S.; Heerwig, A.; Zizak, I.; Kaskel, S.; Walton, R. I.; de Vos, D.; Llewellyn, P.; Lieb, A.; Maurin, G.; Stock, N., Conformation-Controlled Sorption Properties and Breathing of the Aliphatic Al-MOF [Al(OH)(CDC)]. *Inorganic Chemistry* **2014,** *53* (9), 4610-4620.
18. Wang, P.; Kajiwara, T.; Otake, K.-i.; Yao, M.-S.; Ashitani, H.; Kubota, Y.; Kitagawa, S., Xylene Recognition in Flexible Porous Coordination Polymer by Guest-Dependent Structural Transition. *ACS Applied Materials & Interfaces* **2021,** *13* (44), 52144-52151.
19. Kumar, N.; Wang, S.-Q.; Mukherjee, S.; Bezrukov, A. A.; Patyk-Kaźmierczak, E.; O'Nolan, D.; Kumar, A.; Yu, M.-H.; Chang, Z.; Bu, X.-H.; Zaworotko, M. J., Crystal engineering of a rectangular sql coordination network to enable xylenes selectivity over ethylbenzene. *Chemical Science* **2020,** *11* (26), 6889-6895.
20. Gao, M.-Y.; Wang, S.-Q.; Bezrukov, A. A.; Darwish, S.; Song, B.-Q.; Deng, C.; Matos, C. R. M. O.; Liu, L.; Tang, B.; Dai, S.; Yang, S.; Zaworotko, M. J., Switching Adsorbent Layered Material that Enables Stepwise Capture of C8 Aromatics via Single-Crystal-to-Single-Crystal Transformations. *Chemistry of Materials* **2023,** *35* (23), 10001-10008.
21. Jin, Z.; Zhao, H.-Y.; Zhao, X.-J.; Fang, Q.-R.; Long, J. R.; Zhu, G.-S., A novel microporous MOF with the capability of selective adsorption of xylenes. *Chemical Communications* **2010,** *46* (45), 8612-8614.
22. Mukherjee, S.; Joarder, B.; Manna, B.; Desai, A. V.; Chaudhari, A. K.; Ghosh, S. K., Framework-Flexibility Driven Selective Sorption of p-Xylene over Other Isomers by a Dynamic Metal-Organic Framework. *Scientific Reports* **2014,** *4* (1), 5761.
23. Kałuża, A. M.; Mukherjee, S.; Wang, S.-Q.; O'Hearn, D. J.; Zaworotko, M. J., [Cu(4-phenylpyridine)4(trifluoromethanesulfonate)2], a Werner complex that exhibits high selectivity for o-xylene. *Chemical Communications* **2020,** *56* (13), 1940-1943.
24. Warren, J. E.; Perkins, C. G.; Jelfs, K. E.; Boldrin, P.; Chater, P. A.; Miller, G. J.; Manning, T. D.; Briggs, M. E.; Stylianou, K. C.; Claridge, J. B.; Rosseinsky, M. J., Shape Selectivity by Guest-Driven Restructuring of a Porous Material. *Angewandte Chemie International Edition* **2014,** *53* (18), 4592-4596.

25. Lin, J.-M.; He, C.-T.; Liao, P.-Q.; Lin, R.-B.; Zhang, J.-P., Structural, energetic and dynamic insights into the abnormal xylene separation behavior of hierarchical porous crystal. *Scientific Reports* **2015,** *5* (1), 11537.
26. Lusi, M.; Barbour, L. J., Solid–Vapor Sorption of Xylenes: Prioritized Selectivity as a Means of Separating All Three Isomers Using a Single Substrate. *Angewandte Chemie International Edition* **2012,** *51* (16), 3928-3931.
27. Alaerts, L.; Kirschhock, C. E. A.; Maes, M.; van der Veen, M. A.; Finsy, V.; Depla, A.; Martens, J. A.; Baron, G. V.; Jacobs, P. A.; Denayer, J. F. M.; De Vos, D. E., Selective Adsorption and Separation of Xylene Isomers and Ethylbenzene with the Microporous Vanadium(IV) Terephthalate MIL-47. *Angewandte Chemie International Edition* **2007,** *46* (23), 4293-4297.
28. Gee, J. A.; Zhang, K.; Bhattacharyya, S.; Bentley, J.; Rungta, M.; Abichandani, J. S.; Sholl, D. S.; Nair, S., Computational Identification and Experimental Evaluation of Metal–Organic Frameworks for Xylene Enrichment. *The Journal of Physical Chemistry C* **2016,** *120* (22), 12075-12082.
29. Trens, P.; Belarbi, H.; Shepherd, C.; Gonzalez, P.; Ramsahye, N. A.; Lee, U. H.; Seo, Y.-K.; Chang, J.-S., Adsorption and separation of xylene isomers vapors onto the chromium terephthalate-based porous material MIL-101(Cr): An experimental and computational study. *Microporous and Mesoporous Materials* **2014,** *183*, 17-22.
30. Vermoortele, F.; Maes, M.; Moghadam, P. Z.; Lennox, M. J.; Ragon, F.; Boulhout, M.; Biswas, S.; Laurier, K. G. M.; Beurroies, I.; Denoyel, R.; Roeffaers, M.; Stock, N.; Düren, T.; Serre, C.; De Vos, D. E., p-Xylene-Selective Metal–Organic Frameworks: A Case of Topology-Directed Selectivity. *Journal of the American Chemical Society* **2011,** *133* (46), 18526-18529.
31. Gu, Z.-Y.; Jiang, D.-Q.; Wang, H.-F.; Cui, X.-Y.; Yan, X.-P., Adsorption and Separation of Xylene Isomers and Ethylbenzene on Two Zn–Terephthalate Metal–Organic Frameworks. *The Journal of Physical Chemistry C* **2010,** *114* (1), 311-316.
32. Nicolau, M. P. M.; Bárcia, P. S.; Gallegos, J. M.; Silva, J. A. C.; Rodrigues, A. E.; Chen, B., Single- and Multicomponent Vapor-Phase Adsorption of Xylene Isomers and Ethylbenzene in a Microporous Metal–Organic Framework. *The Journal of Physical Chemistry C* **2009,** *113* (30), 13173-13179.
33. Peralta, D.; Chaplais, G.; Paillaud, J.-L.; Simon-Masseron, A.; Barthelet, K.; Pirngruber, G. D., The separation of xylene isomers by ZIF-8: A demonstration of the extraordinary flexibility of the ZIF-8 framework. *Microporous and Mesoporous Materials* **2013,** *173*, 1-5.
34. Moreira, M. A.; Santos, J. C.; Ferreira, A. F. P.; Loureiro, J. M.; Ragon, F.; Horcajada, P.; Shim, K.-E.; Hwang, Y.-K.; Lee, U. H.; Chang, J.-S.; Serre, C.; Rodrigues, A. E., Reverse Shape Selectivity in the Liquid-Phase Adsorption of Xylene Isomers in Zirconium Terephthalate MOF UiO-66. *Langmuir* **2012,** *28* (13), 5715-5723.

35. Peralta, D.; Barthelet, K.; Pérez-Pellitero, J.; Chizallet, C.; Chaplais, G.; Simon-Masseron, A.; Pirngruber, G. D., Adsorption and Separation of Xylene Isomers: CPO-27-Ni vs HKUST-1 vs NaY. *The Journal of Physical Chemistry C* **2012,** *116* (41), 21844-21855.
36. Gonzalez, M. I.; Kapelewski, M. T.; Bloch, E. D.; Milner, P. J.; Reed, D. A.; Hudson, M. R.; Mason, J. A.; Barin, G.; Brown, C. M.; Long, J. R., Separation of Xylene Isomers through Multiple Metal Site Interactions in Metal–Organic Frameworks. *Journal of the American Chemical Society* **2018,** *140* (9), 3412-3422.
37. Torres-Knoop, A.; Krishna, R.; Dubbeldam, D., Separating Xylene Isomers by Commensurate Stacking of p-Xylene within Channels of MAF-X8. *Angewandte Chemie International Edition* **2014,** *53* (30), 7774-7778.
38. Huang, W.; Jiang, J.; Wu, D.; Xu, J.; Xue, B.; Kirillov, A. M., A Highly Stable Nanotubular MOF Rotator for Selective Adsorption of Benzene and Separation of Xylene Isomers. *Inorganic Chemistry* **2015,** *54* (22), 10524-10526.
39. Li, X.; Wang, J.; Bai, N.; Zhang, X.; Han, X.; da Silva, I.; Morris, C. G.; Xu, S.; Wilary, D. M.; Sun, Y.; Cheng, Y.; Murray, C. A.; Tang, C. C.; Frogley, M. D.; Cinque, G.; Lowe, T.; Zhang, H.; Ramirez-Cuesta, A. J.; Thomas, K. M.; Bolton, L. W.; Yang, S.; Schröder, M., Refinement of pore size at sub-angstrom precision in robust metal–organic frameworks for separation of xylenes. *Nature Communications* **2020,** *11* (1), 4280.
40. Yang, L.; Liu, H.; Xing, J.; Yuan, D.; Xu, Y.; Liu, Z., Separation of Xylene Isomers in the Anion-Pillared Square Grid Material SIFSIX-1-Cu. *Chemistry – A European Journal* **2021,** *27* (20), 6187-6190.
41. Jie, K.; Liu, M.; Zhou, Y.; Little, M. A.; Pulido, A.; Chong, S. Y.; Stephenson, A.; Hughes, A. R.; Sakakibara, F.; Ogoshi, T.; Blanc, F.; Day, G. M.; Huang, F.; Cooper, A. I., Near-Ideal Xylene Selectivity in Adaptive Molecular Pillar[n]arene Crystals. *Journal of the American Chemical Society* **2018,** *140* (22), 6921-6930.
42. Rasouli, M.; Yaghobi, N.; Chitsazan, S.; Sayyar, M. H., Influence of monovalent cations ion-exchange on zeolite ZSM-5 in separation of para-xylene from xylene mixture. *Microporous and Mesoporous Materials* **2012,** *150*, 47-54.
43. Rasouli, M.; Yaghobi, N.; Allahgholipour, F.; Atashi, H., Para-xylene adsorption separation process using nano-zeolite Ba-X. *Chemical Engineering Research and Design* **2014,** *92* (6), 1192-1199.
44. Rasouli, M.; Yaghobi, N.; Movassaghi Gilani, S. Z.; Atashi, H.; Rasouli, M., Influence of monovalent alkaline metal cations on binder-free nano-zeolite X in para-xylene separation. *Chinese Journal of Chemical Engineering* **2015,** *23* (1), 64-70.
